# In Situ Synthesis and Applications for Polyinterhalides Based on BrCl

**DOI:** 10.1002/chem.202001267

**Published:** 2020-10-22

**Authors:** Benjamin Schmidt, Sebastian Ponath, Johannes Hannemann, Patrick Voßnacker, Karsten Sonnenberg, Mathias Christmann, Sebastian Riedel

**Affiliations:** ^1^ Fachbereich Biologie, Chemie, Pharmazie Institut für Chemie und Biochemie—Anorganische Chemie Fabeckstr. 34/36 14195 Berlin Germany; ^2^ Fachbereich Biologie, Chemie, Pharmazie Institut für Chemie und Biochemie—Organische Chemie Takustr. 3 14195 Berlin Germany

**Keywords:** bromine monochloride, halogen bonding, halogenation, polyhalides

## Abstract

The use of neat BrCl in organic and inorganic chemistry is limited due to its gaseous aggregate state and especially its decomposition into Cl_2_ and Br_2_. The stabilization of BrCl in form of reactive ionic liquids via a novel in situ synthesis route shifts this equilibrium drastically to the BrCl side, which leads to safer and easier‐to‐handle interhalogenation reagents. Furthermore, the crystalline derivatives of the hitherto unknown [Cl(BrCl)_2_]^−^ and [Cl(BrCl)_4_]^−^ anions were synthesized and characterized by single‐crystal X‐ray diffraction (XRD), Raman and IR spectroscopy, as well as quantum chemical calculations.

## Graphical abstract

**A new synthesis route** enables the stabilization of the toxic gas BrCl in form of reactive ionic liquids (ILs). With these ILs it is possible to interhalogenate Michael systems, alkenes and alkynes in fast reaction times. Furthermore a complete set of the possible BrCl interhalides with one to six coordinating BrCl molecules was synthesized, including the hitherto unknown [Cl(BrCl)_2_]^−^ and [Cl(BrCl)_4_]^−^ anions.

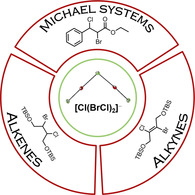

## Introduction

After the discovery of attractive interactions between dihalogens and halide anions in 1819,^[1]^ Chattaway and Hoyle carried out first systematic investigations of polybromides and ‐chlorides in 1923.^[2]^ These reports marked the starting point of the vast chemistry of polyhalogen compounds. In the last years not only our knowledge of the structural diversity of several polyhalogen anions, but also the possible applications of polyhalogen compounds increased continuously.^[3]^ In general, interhalogen anions can be separated into classical interhalides, which possess an electropositive center that is surrounded by more electronegative halogen atoms, for example, [ICl_4_]^−^,^[4]^ [IF_6_]^−^,^[5]^ and nonclassical interhalides. A nonclassical interhalide can be best described as a central halide ion X^−^, which is surrounded by dihalogen Y_2_ (e.g., [Cl(I_2_)_4_]^−[6]^) or interhalogen molecules XY/YZ (e.g., [Br(IBr)_2_⋅2 IBr]^−^).^[7]^ Lately first examples of extraordinarily large polyhalogen anions of the lighter halogens such as the octahedrally coordinated monoanions [Cl_13_]^−[8]^ and [Cl(BrCl)_6_]^−[9]^ as well as the highly reactive [Br_2_F_7_]^−^ and [Br_3_F_10_]^−^ anions^[10]^ were reported. Besides the detailed structural investigation of polyhalides, their applications as halogenation reagents,^[11]^ as electrolytes in dye‐sensitized solar cells^[12]^ or (redox flow) batteries^[13]^ are well established. Recently further applications as reactive ionic liquids which are able to dissolve noble metals and alloys were reported. These reactive ionic liquids show promise for applications in metal recycling.^[14]^

**Figure 1:**
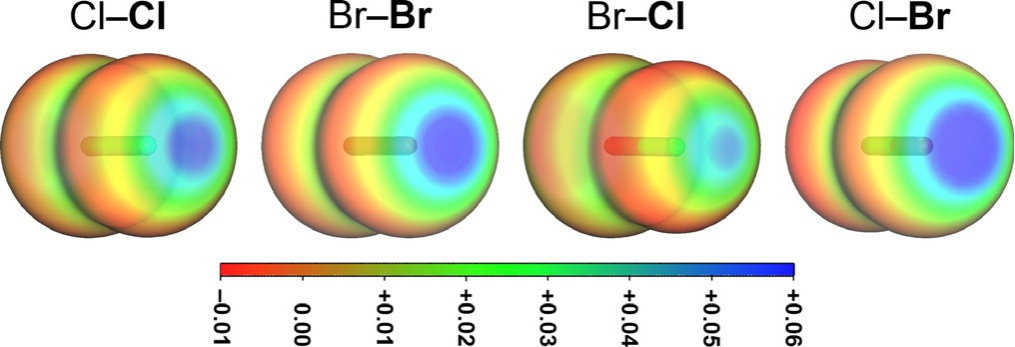
Electrostatic potential in the range of −0.01 au (red) to 0.06 au (blue) for the molecules Cl_2_, Br_2_ and BrCl (view of the **Cl** or **Br** atom, isosurface value 0.0035 au); calculated at the B3LYP‐D3/def2‐TZVPP level of theory.

The formation of polyhalides can be explained by the concept of halogen bonding. According to this concept, the electrostatic potential of a dihalogen molecule is anisotropic and can be divided into two regions: an area of higher electron density, which forms a belt perpendicular to the molecule's bonding axis, and a region of a more positive electrostatic potential, which is situated on the bonding axis, the so‐called σ‐hole.^[15]^ While for symmetrical dihalogens such as Br_2_ and Cl_2_, the σ‐hole is symmetrical on both halogen atoms, for diatomic interhalogens such as IBr and BrCl, the σ‐hole is more pronounced at the more electropositive halogen atom, see Figure 1. Owing to their polarized bond and pronounced σ‐hole, interhalides are expected to form more stable anions and their tendency to form extended polyhalogen networks is lowered.

## Results and Discussion

**Scheme 1:**
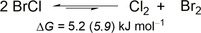
Equilibrium of BrCl with Cl_2_ and Br_2_ under ambient conditions; Δ*G* (298.15 K, 0.1 MPa) values were calculated at B3LYP‐D3(BJ)/def2‐TZVPP and SCS‐MP2/def2‐TZVPP (*italic*) level of theory.

Considering the excellent halogen bonding properties, it is surprising that larger nonclassical interhalides based on bromine monochloride (BrCl) have been reported only recently.^[9]^ The interhalide BrCl exists in an equilibrium with Br_2_ and Cl_2_, see Scheme 1.[^16^, ^17^] This hampers its use as a reagent due to side reactions. An equilibrium ratio of approximately 60 % for BrCl, and 20 % for Cl_2_ and 20 % Br_2_ was experimentally determined at room temperature.^[16]^

**Scheme 2:**
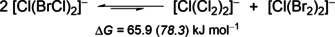
Equilibrium of stabilized BrCl under ambient conditions; Δ*G* (298.15 K, 0.1 MPa) values were calculated at B3LYP‐D3(BJ)/def2‐TZVPP and SCS‐MP2/def2‐TZVPP (*italic*) level of theory.

This in good agreement with quantum chemical calculations at B3LYP‐D3(BJ)/def2‐TZVPP^[18]‐[28]^ and SCS‐MP2/def2‐TZVPP[^29^, ^30^] level of theory. This equilibrium makes the stoichiometric usage of neat BrCl nearly impossible. Due to the different vapor pressures^[31]^ of Br_2_, Cl_2_ and BrCl, first mainly chlorine boils off a batch of BrCl while bromine is enriched in the remaining liquid. Due to the pronounced σ‐hole, the halogen bonding properties of BrCl are enhanced in comparison to Br_2_ and Cl_2_. Thus, an addition of a halogen bond acceptor, for example, a chloride salt, yielding BrCl based polyinterhalides such as the pentahalide [Cl(BrCl)_2_]^−^, should result in a significant shift of the equilibrium to the BrCl side, see Scheme 2. Our calculations show that the equilibrium is almost entirely located on the side of the BrCl based interhalides (>99.99 %, calculated from Δ*G°*). Therefore, the stabilization of BrCl in form of polyinterhalide compounds provides a convenient BrCl source.

This was partially examined by the use of the trihalide [Cl(BrCl)]^−^ as efficient bromochlorinating agent.[^32^, ^33^, ^34^] Nevertheless, further studies regarding the reactivity of higher polyinterhalides based on BrCl are still necessary.

Herein; we report a new synthetic route for polyinterhalides based on BrCl as well as the preparation and characterization of hitherto unknown BrCl interhalide monoanions, which complete the set of possible coordination numbers. These compounds have been characterized by single crystal X‐ray diffraction (XRD), Raman and IR spectroscopy as well as quantum chemical calculations.

**Scheme 3:**
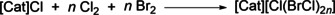
In situ synthesis of interhalogen compounds based on BrCl.

A new synthetic approach generates the interhalide BrCl in situ, see Scheme 3. In this approach, a chloride salt was provided in a reaction flask and afterwards elemental chlorine and bromine were condensed onto the salt at −196 °C.

Condensing Br_2_ and Cl_2_ as starting materials guarantees the exact stoichiometry compared to the usage of a previously prepared BrCl batch. Warming up to ambient temperature leads to the formation of BrCl, which immediately reacts with the chloride to afford the desired polyinterhalide species. Due to this in situ generation of BrCl the undesired impurities of Br_2_ and Cl_2_ are minimized and consequently a clean product is obtained. These interhalide compounds offer themselves as an easy‐to‐handle and safer alternative to neat BrCl for applications in organic and inorganic chemistry.

**Figure 2:**
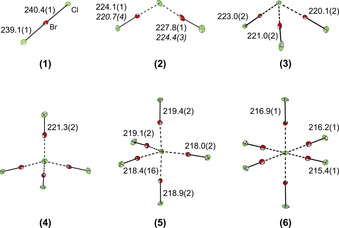
Molecular structures in the solid state of the interhalide salts [Cat][Cl(BrCl)_*n*_] (for *n=*1, 2, 3, 5 [Cat]^+^=[NEt_4_]^+^; for *n=*4 [Cat]^+^=[NPr_4_]^+^; for *n=*6 [Cat]^+^=[PNP]^+ [9]^) with thermal ellipsoids set at 50 % probability. The cations and disorders of the anions were omitted for clarity. For the pentahalides (**2**) two different structures, namely [NEt_4_]_2_[Cl(BrCl)_2_][Cl(BrCl)] and[NEt_4_][Cl(BrCl)_2_] (bond lengths in *italics*), were obtained. For detailed structures, see Figures S1–S5, S38. Bond lengths [pm] of the central chloride to the coordinated BrCl molecules: **2**: 251.7(1), 262.3(1); 259.6(1), 265.5(1); **3**: 266.4(2)–273.0(2); **4**: 278.6(1); **5**: 281.1(2)–298.6(2); **6**: 279.3(1)–291.3(1).

To demonstrate the advantages of the new synthesis route, a complete set of the possible BrCl interhalides with one to six coordinating BrCl molecules was synthesized and fully characterized, see Figure 2.

To receive the trihalide [Cl(BrCl)]^−^, 0.5 equivalents of Br_2_ and Cl_2_ were condensed onto [NEt_4_]Cl in dichloromethane (DCM). Slowly cooling the reaction mixture to −24 °C yields single crystals suitable for XRD analysis. The obtained crystal structure of **1** was already published in 1974.^[35]^ The structural motif of the [Cl(BrCl)]^−^ trihalide was discussed several times in literature.^[36]^ [NEt_4_][Cl(BrCl)] contains two crystallographically independent anions in the asymmetric unit. In the following, the structural data of the more symmetrical anion is discussed. The trihalide is almost linear (179.1(1)°) and symmetrical (Cl1−Br1 240.4(1), Cl2−Br1 239.1(1) pm). The bonding situation can be best described as a three‐center four‐electron (3c−4e) bond and can be compared with other symmetrical trihalides such as [Br_3_]^−^ or [Cl_3_]^−^.[^3^, ^37^] The 3c‐4e bond results in a bond order of 0.5, which explains the most elongated BrCl bond in comparison to the larger interhalides.

The addition of 0.85 equivalents of the dihalogens Br_2_ and Cl_2_ to [NEt_4_]Cl in DCM leads to the formation of the hitherto unknown pentahalide [Cl(BrCl)_2_]^−^. The anion possesses a slightly distorted V‐shaped structure, see Figure S38. In the crystal structure a partial substitution of the coordinating BrCl molecules by Br_2_ is observed. Therefore, a small excess of chlorine (0.85 equiv Br_2_, 1.1 equiv Cl_2_) was used resulting in crystals of a non‐disordered [Cl(BrCl)_2_]^−^ anion and an additional, slightly unsymmetrical [Cl(BrCl)]^−^, see Figure S2. In contrast to the 3c‐4e bond of trihalides, the bonding situation of the higher poly(inter)halides can be best described as a donor–acceptor interaction. The central halide ion acts as Lewis base and donates electron density into the LUMO (σ*) of the Lewis acids, namely the coordinating dihalogen molecules. The charge transfer results in a bond weakening and elongation of the coordinating molecules, which can also be observed experimentally.^[3]^ In case of the non‐disordered pentahalide **2** the bond lengths of the coordinating BrCl molecules are elongated by 10.5(2)–14.2(2) pm compared to free BrCl (213.6(1) pm).^[38]^ Adding more equivalents of Br_2_ and Cl_2_ to the chloride salt, anions with higher coordination numbers can be obtained. Consequently the heptahalide [Cl(BrCl)_3_]^−^ (**3**) crystallized with [NEt_4_]^+^ as the counter ion. The heptahalide was already reported with [AsPh_4_]^+^ as the cation. In the reported structure of [AsPh_4_][Cl(BrCl)_3_] two crystallographically independent heptahalides, which slightly interact with each other, can be observed in the asymmetric unit.^[9]^ The [Cl(BrCl)_3_]^−^ presented here in [NEt_4_][Cl(BrCl)_3_] consists of just one discrete anion, which shows no interactions to other halogen atoms and is arranged in a distorted trigonal‐pyramidal structure.

**Figure 3:**
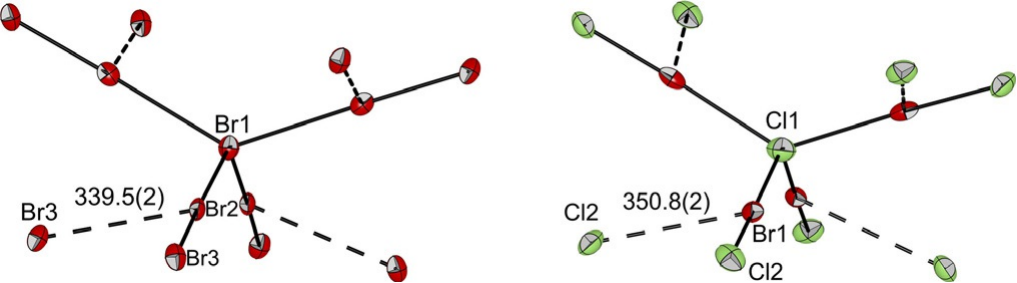
Comparison of the anion structures in the solid state of [NPr_4_][Br(Br_2_)_4_]^*−*^ and [NPr_4_][Cl(BrCl)_4_]^*−*^ with thermal ellipsoids set at 50 *%* probability. Selected bond lengths [pm] and angles [°]: [Br_9_]^*−*^: Br1−Br2 294.3(1), Br2−Br1‐Br2′ 131.2(1), 99.8(1); [Cl(BrCl)_4_]^*−*^: Cl1−Br1 278.6(1), Br1‐Cl1‐Br1′ 130.1(1), 100.3(1).

Increasing the amount of Br_2_ and Cl_2_ to 2.5 equivalents and changing the cation to [NPr_4_]^+^ leads to the formation of the nonahalide [Cl(BrCl)_4_]^−^, which is the first reported BrCl based nonahalide, see Figure 3.

The compound crystallizes in the tetragonal space group *I*_4_ and its structure can be best described as a distorted tetrahedron. The obtained polyinterhalide compounds is isostructural to the already published [NPr_4_][Br(Br_2_)_4_] (Figure 3).^[39]^ In comparison to the [Br(Br_2_)_4_]^−^, whose intermolecular distances are about 30 pm shorter than the sum of the van der Waals radii (370 pm),^[40]^ the [Cl(BrCl)_4_]^−^ anion is more discrete. The intermolecular distances of **4** are just 10 pm shorter than the sum of the van der Waals radii (360 pm),^[40]^ which again shows the lower tendency of BrCl based interhalides to be stabilized by secondary halogen‐halogen interactions. Furthermore, the hypothetical square‐planar structure (*D*_4*h*_) has been quantum‐chemically investigated at B3LYP‐D3(BJ)/def2‐TZVPP and SCS‐MP2/def2‐TZVPP level. Both optimized structures show a transition state for the interconversion of the tetrahedral structure which are 14.6 and 7.2 kJ mol^−1^ higher in energy than the *T*_d_ structure.

**Figure 4:**
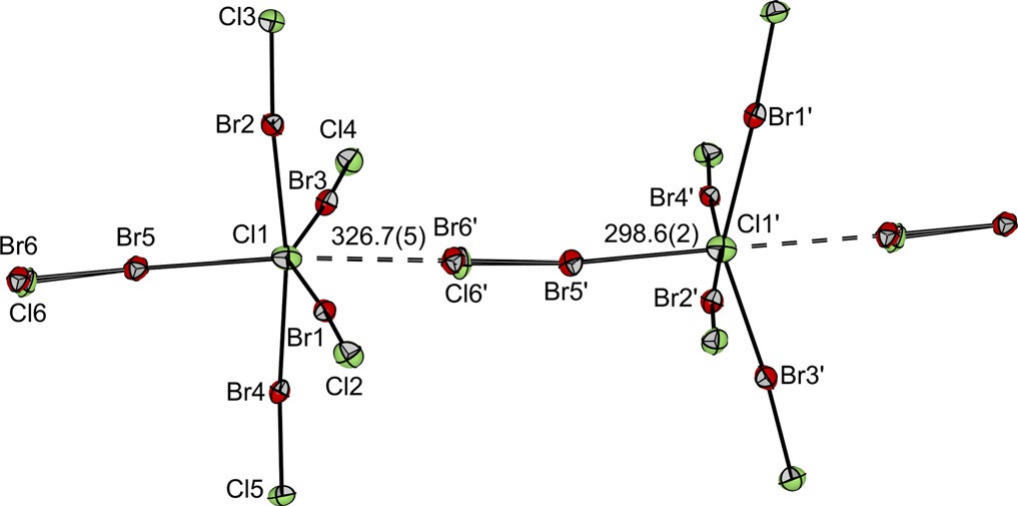
Molecular structures in the solid state of the [Cl(BrCl)_5_]^−^ anions in [NEt_4_][Cl(BrCl)_5_] with thermal ellipsoids set at 50 *%* probability. A Br_2_/BrCl (Br5‐Br6/Br5‐Cl6: 60 *%*/40 *%* occupation) interacts with two central chloride ions to form a chain (*d*(Cl1−Br6′)=326.7(5) pm, *d*(Cl1−Cl6′)=340.6(17) pm). Selected bond lengths [pm] and angles [°]: Cl1−Br1 286.4(2), Cl1−Br2 281.6(2), Cl1−Br3 281.4(2), Cl1−Br4 281.1(2), Cl1−Br5 298.6(2), Br1−Cl2 218.0(2), Br2−Cl3 219.4(2), Br3−Cl4 219.1(2), Br4−Cl5 218.9(2), Br5−Cl6 218.4(16), Br5−Br6 231.4(5); Cl1‐Br1‐Cl2 176.7(1), Cl1‐Br2‐Cl3 174.9(1), Cl1‐Br3‐Cl4 175.3(1), Cl1‐Br4‐Cl5 176.1(1), Cl1‐Br5‐Cl6 174.8(8), Br3‐Cl1‐Br1 148.7(1), Br2‐Cl1‐Br4 171.5(1).

From a reaction mixture of an excess of Br_2_ and Cl_2_ (3.5 equiv each) with [NEt_4_]Cl, single crystals of the undecainterhalide [NEt_4_][Cl(BrCl)_5_] were obtained (**5**). A similar undecahalide structure was already published earlier with [CCl(NMe_2_)_2_]^+^ as the counter ion. With this cation the structural parameter *τ* (*τ*=(*β*−*α*)/60°, *β* and *α* are the largest angles in the coordination sphere) was determined to be *τ*=0.25, which indicated a rather square‐pyramidal arrangement.[^9^, ^41^] With [NEt_4_]^+^ as the cation, the structural parameter becomes larger (*τ*=0.38). This underlines the strong influence of the cation on the structure of the anion. Another reason for the distorted structure of the undecahalide is the bridging between the [Cl(BrCl)_5_]^−^ units, which leads to an octahedral coordination sphere for the central chloride Cl1, see Figure 4.

As mentioned above, the tendency to form extended networks is lowered for BrCl due to its asymmetrical σ‐hole in comparison to the symmetrical Br_2_. The solid state structure of [Cl(BrCl)_5_]^−^ (**5**) possesses one disordered coordinating dihalogen unit (Br5–Cl6/Br6), which is bridging between two anions, see Figure 4. The interaction of the bromine with the two central chloride ions leads to the formation of a chain.

It was possible to synthesize and crystallize interhalides based on BrCl with tetraalkylammonium as the cation with coordination numbers going from one up to five. The hexa‐coordinated tridecainterhalide could not be isolated with tetraalkylammonium cations, even an excess of BrCl leads to the formation of the [Cl(BrCl)_5_]^−^ anion. This underlines the great influence of the counter ions size on the obtained anion. Using the large bis(triphenylphosphoranylidene)iminium ([PNP]^+^) as the cation leads to the formation of the tridecainterhalide, which was already published.^[9]^ In this series of polyinterhalides the Br–Cl bond length of the terminal BrCl molecules is decreasing with the increase of BrCl molecules coordinating to the central atom.

As mentioned before, the [Cl(BrCl)]^−^ anion exhibits the longest BrCl bond due to the 3c‐4e bond. From the pentahalide to the tridecahalide the bonds of the coordinating BrCl molecules are less weakened and therefore less elongated compared to the trihalide and free BrCl (213.6(1) pm).^[38]^ The bonding situation of higher polyinterhalides can be described as donor/acceptor interaction between the central chloride ion, the Lewis base, and the surrounding BrCl molecules, which function as Lewis acids.

**Figure 5:**
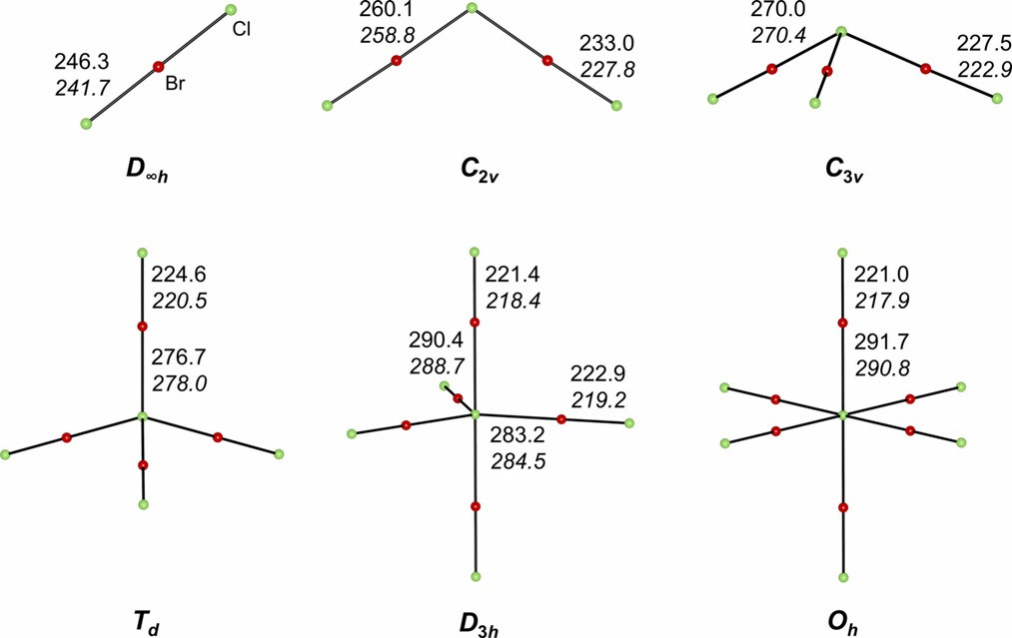
Optimized minima structures of [Cl(BrCl)_*n*_]^−^ (*n*=1*–*6). Calculated bond lengths in pm at the B3LYP‐D3(BJ)/def2‐TZVPP level (SCS‐MP2/def2‐TZVPP level in italics) are indicated.

The higher the coordination number of the central chloride the less electron density can be donated into each LUMO (σ*) of each coordinating BrCl molecule. This leads to less weakening of the BrCl bond accompanied with shorter bond lengths, see Figure 2. Quantum chemical calculations for the minima structures in the gas phase of the polyinterhalide species (at the B3LYP‐D3(BJ)/def2‐TZVPP and SCS‐MP2/def2‐TZVPP level of theory) are in good agreement with the experimental data and verify the described trend, see Figure 5.

**Figure 6:**
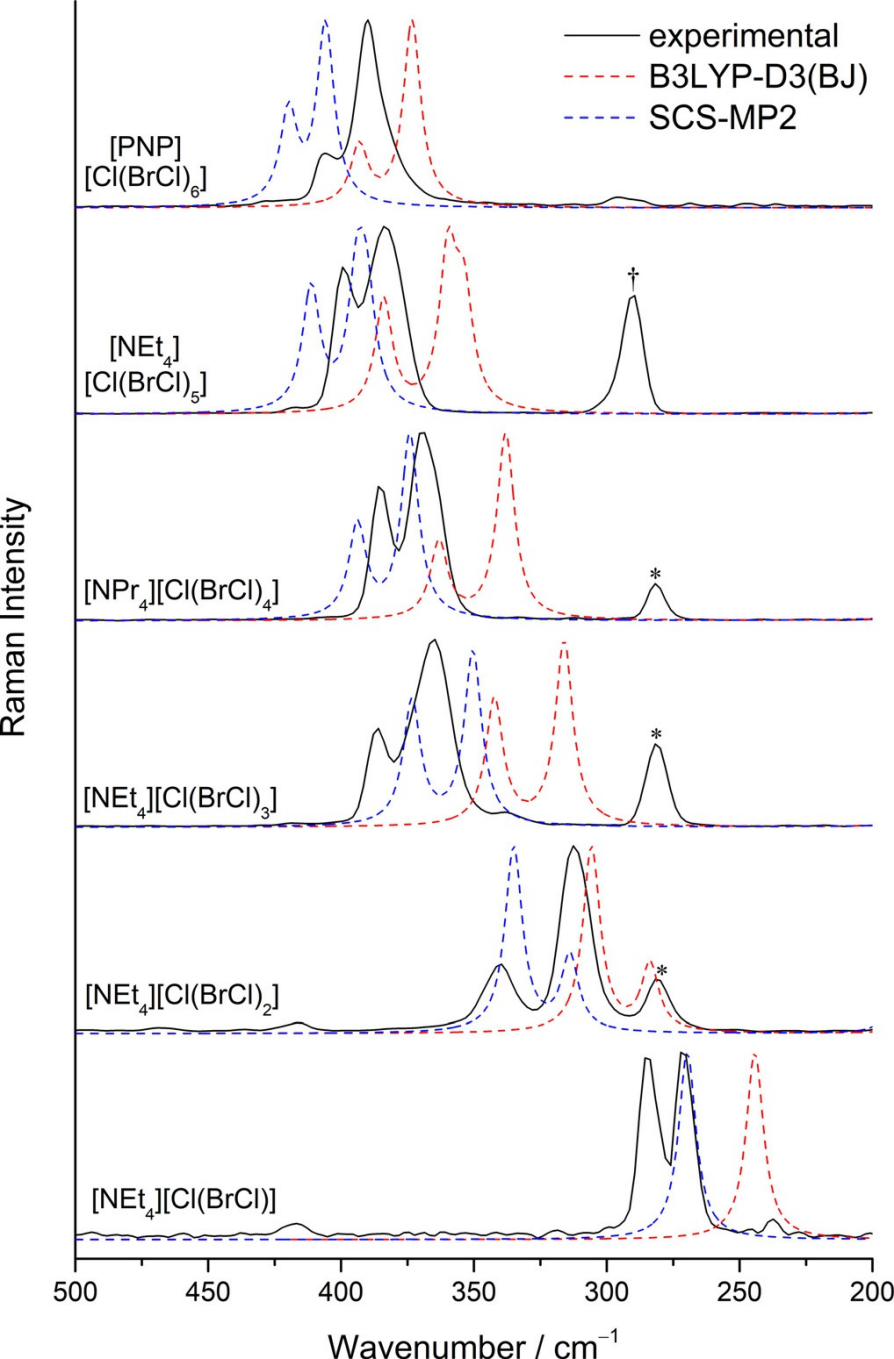
Low temperature Raman spectra of single crystals of the polyinterhalide salts (black) as well as calculated Raman spectra at the SCS‐MP2/def2‐TZVPP (blue) and B3LYP‐D3(BJ)/def2‐TZVPP (red) level of theory. The band at 281 cm^−1^ associated with [Cl(BrCl)]^−^ is indicated by asterisks; the band for the bridging Br_2_ molecule is indicated by a dagger.

Due to their strong Raman scattering, Raman spectroscopy is highly instructive for further analysis of polyinterhalide compounds. Therefore, low temperature Raman spectra of single crystals of each polyinterhalide salt were recorded and compared with quantum chemical calculations, see Figure 6. The trend of decreasing bond weakening of the BrCl units leads to a shift of the BrCl stretching frequencies to higher wavenumbers in the corresponding Raman spectra. Due to the almost inversion‐symmetrical structure of trihalides, only the symmetric stretching vibration possesses significant Raman intensity. In the spectrum of [NEt_4_]_2_[Cl(BrCl)]_2_, two bands can be observed at 285 and 271 cm^−1^ respectively. These two bands can be explained by two symmetric stretching vibrations of two crystallographically independent [Cl(BrCl)]^−^ anions, which differ in bond lengths and angles (241.8(1), 235.7(1) pm, 174.8(1)°; 240.4(1), 239.1(1) pm, 179.1(1)°). These bands are most shifted to lower wavenumbers in comparison to free BrCl (434 cm^−1^)^[42]^ due to the 3c−4e bond in the trihalide. Quantum chemical calculations for the trihalide in *D*_∞*h*_ symmetry at the SCS‐MP2/def2‐TZVPP level of theory predict one band for the symmetrical stretching vibration at 270 cm^−1^ (*A*_1*g*_), which agrees well with the experimental results.

As described above, the bond weakening decreases with increasing number of coordinating BrCl molecules. The corresponding bands shift to higher wavenumbers and almost reach the frequency of free BrCl. In case of the larger polyinterhalide compounds two major bands in the Br–Cl stretching region can be observed, the symmetric and asymmetric stretch of the coordinating BrCl molecules.

In the spectra of the three and four times coordinated interhalogen compounds, an additional band at 281 cm^−1^ is observed, which can be assigned to a [Cl(BrCl)]^−^ species, that results from residual mother liquor. The stretching frequency of the bridging Br_2_ molecule in the solid state structure of the undecainterhalide **5** is observed at 290 cm^−1^. In the Raman spectrum of the pentainterhalide **2**, the intensities of the symmetric and asymmetric stretching modes are inverted with respect to the calculations. This can be explained by interactions in the solid state, as shown by Raman spectra of compound **2** in a DCM solution and in bulk, see Figure S42. All experimental spectra are in good agreement with quantum‐chemical calculations, see Figure 6.

Additionally, room‐temperature ionic liquids (RT‐ILs) containing 1–3 equiv of BrCl were prepared and analyzed by IR spectroscopy (Figure S12). The described trend of bond weakening and shift of vibrational frequencies is also visible in the IR spectra, however Raman spectroscopy is more suited to characterize the polyinterhalide species due to the superior signal to noise ratio.

The usage of poly(inter)halide compounds in organic and inorganic synthesis is growing over the last years.

**Scheme 4:**
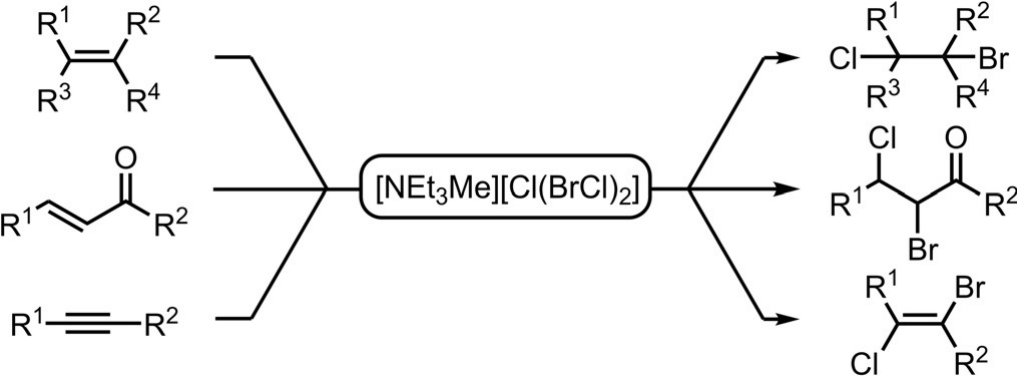
Interhalogenation of alkenes, Michael systems and alkynes using the reactive ionic liquid based on BrCl (for a detailed substrate scope see Table S4).

The bromochlorinating agent [Cl(BrCl)]^−^ was already reported in the 1980s.[^32^, ^33^, ^34^] To demonstrate the reactivity of the polyinterhalide ionic liquid, a variety of substrates including several alkenes, Michael systems and an alkyne was reacted with [NEt_3_Me][Cl(BrCl)_2_], see Scheme 4 (and Supporting Information, chapters f and g).

The reactions were performed in dichloromethane (DCM) at −78 °C to provide the bromochlorinated product within 60 min reaction time in yields between 71 and 91 %. The interhalogenation of several alkenes and Michael systems was achieved even at low temperatures in very short reaction times and good yields. To investigate the long‐term stability of these reactive interhalogenation reagents, Raman spectra of two BrCl based ionic liquids were measured continuously over a period of one year, see Figures S36–37. The prepared ILs ([NMe_4_]Cl+4.5 equiv BrCl, [NPr_4_]Cl+4.5 equiv BrCl) were stored under the exclusion of light in sealed glass ampules. Both ILs are stable against halogenation for at least one year, which proofs that tetraalkylammonium cations ([NR_4_]^+^, R=Me, Et, Pr) are suitable counter ions for these polyinterhalide reagents.

## Conclusions

In summary, we have presented a novel in situ synthesis route for BrCl based interhalides. The addition of a halogen bond acceptor, for example, a chloride salt, results in an almost entire shift of the equilibrium to the BrCl side (>99.99 %). This stabilization of the reactive BrCl provides safer and easy‐to‐handle polyinterhalide ILs for further applications such as interhalogenation reactions of alkenes, alkynes and Michael systems in organic synthesis. Furthermore, we were able to synthesize via this new route a complete set of the possible BrCl interhalides with coordination numbers of one to six coordinating BrCl molecules, showing that the average Cl–Br distances of the central chloride to the surrounding BrCl molecule correlate well with the coordination number. Within this set, the hitherto unknown V‐shaped [Cl(BrCl)_2_]^−^ and the distorted tetrahedral [Cl(BrCl)_4_]^−^ anion were reported for the first time. All structures were characterized by single‐crystal X‐ray diffraction, single‐crystal Raman spectroscopy as well as quantum chemical calculations. Hence, our results not only complete the variety of BrCl based interhalides, they also provide a new syntheses route to stabilize the gaseous BrCl for promising synthetic applications.

## Experimental Section

**Materials, instruments, and methods**: All preparative work was carried out using standard Schlenk techniques. Chlorine (Linde, purity 2.8) was passed through calcium chloride before use to remove traces of water. Bromine (purity >99 %) was distilled and stored over activated molecular sieve. The [NR_4_]Cl salts were obtained from commercial sources, dried for two days at 80 °C at reduced pressure and stored under inert conditions. Dichloromethane was stored over activated 3 Å molecular sieve. Raman spectra were recorded on a Bruker (Karlsruhe, Germany) MultiRAM II equipped with a low‐temperature Ge detector (1064 nm, 60 mW, resolution of 4 cm^−1^). Spectra of single crystals were recorded at −196 °C using the Bruker RamanScope III. ATR‐IR spectra of the ILs were recorded with a resolution of 4 cm^−1^ on a Thermo Scientific Nicolet iS50 FT‐IR with DTGS‐polyethylene detector for the FIR range and for the halogenated alkene on a JASCO FT/IR‐4100 spectrometer. NMR spectra were recorded at RT on a JEOL Eclipse+ 500 spectrometer. Mass spectroscopy was performed on an Agilent Technologies 6210 ESI‐TOF spectrometer. X‐ray diffraction data were collected on a Bruker D8 Venture CMOS area detector (Photon 100) diffractometer with MoKα radiation, see Table S1–S2.

Deposition numbers 1965314, 1965315, 1965317, 1965318, 1971174, 1984581, and 1984909 contain the supplementary crystallographic data for this paper. These data are provided free of charge by the joint Cambridge Crystallographic Data Centre and Fachinformationszentrum Karlsruhe Access Structures service.

Single crystals were coated with perfluoroether oil at low temperature (−40 °C) and mounted on a 0.2 mm Micromount. The structure was solved with the ShelXT^[43]^ structure solution program using intrinsic phasing and refined with the ShelXL^[44]^ refinement package using least squares on weighted *F2* values for all reflections using OLEX2.^[45]^

On the one hand, structure optimizations of the polyinterhalide anions were performed at DFT level using the RI‐B3LYP hybrid functional with Grimme's dispersion correction D3 and BJ‐damping together with the def2‐TZVPP basis set.^[18]‐[28]^ On the other hand, further optimizations were performed at SCS‐MP2 level using the def2‐TZVPP basis set.[^29^, ^30^] All calculations were carried out using the TURBOMOLE V7.3 program.^[46]^ Minima on the potential energy surface were characterized by harmonic vibrational frequency analyses. Thermochemistry was provided with zero‐point vibrational correction, Δ*G* values were calculated at 298.15 K and 1.0 bar.

**[NEt_4_][Cl(BrCl)]**: Bromine (113 mg, 0.71 mmol, 0.5 equiv), chlorine (50.1 mg, 0.71 mmol, 0.5 equiv) and dichloromethane (0.15 mL) were condensed at −196 °C onto [NEt_4_]Cl (234 mg, 1.41 mmol, 1.0 equiv). Thawing to room temperature and slowly cooling to −24 °C resulted in the formation of crystals of [NEt_4_][Cl(BrCl)]. Raman (1064 nm, 77 K, 150 mW): 3000(w), 2993(w), 2985(w), 2943(m), 1472(vw), 1457(vw), 1439(vw), 1299(vw), 1124(vw), 1001(vw), 917(vw), 734(vw), 674(w), 417(vw), 285(vs., *ν*_s_ Br–Cl), 271(vs., *ν*_s_ Br–Cl), 238(vw) cm^−1^.

**[NEt_4_]_2_[Cl(BrCl)_2_][Cl(BrCl)]**: Bromine (225 mg, 1.411, mmol, 0.85 equiv), chlorine (129 mg, 1.82 mmol, 1.1 equiv) and dichloromethane (0.20 mL) were condensed at −196 °C onto [NEt_4_]Cl (325 mg, 1.66 mmol, 1.0 equiv). Thawing to room temperature and slowly cooling to −24 °C resulted in the formation of crystals of [NEt_4_][Cl(BrCl)_2_]. Raman (1064 nm, 77 K, 150 mW): $\tilde{\nu }$ = 3013(vw), 2990(w), 2983(w), 2939(w), 1470(vw), 1457(vw), 1300(vw), 1123(vw), 998(vw), 890(vw), 673(vw), 468(vw), 416(w), 340(m, *ν*_s_ Br–Cl), 312(vs., *ν*_as_ Br–Cl), 281(m, *ν*_s_ Br–Cl of [Cl(BrCl)]^−^), 190(w), 158(w), 133(w) cm^−1^.

**[NEt_4_][Cl(BrCl)_3_]**: Bromine (363 mg, 2.27 mmol, 1.5 equiv), chlorine (161 mg, 2.27 mmol, 1.5 equiv) and dichloromethane (0.15 mL) were condensed at −196 °C onto [NEt_4_]Cl (251 mg, 1.51 mmol, 1.0 equiv). Thawing to room temperature and slowly cooling to −24 °C resulted in the formation of crystals of [NEt_4_][Cl(BrCl)_3_]. Raman (1064 nm, 77 K, 150 mW): $\tilde{\nu }$ = 2992(vw), 2981(vw), 2952(vw), 2938(vw), 1456(vw), 1299(vw), 1120(vw), 1000(vw), 679(vw), 493(vw), 387(m, *ν*_s_ Br–Cl), 365(vs., *ν*_as_ Br–Cl), 281(vs., *ν*_s_ Br–Cl of [Cl(BrCl)]^−^), 177(vw), 137(w) cm^−1^.

**[NPr_4_][Cl(BrCl)_4_]**: Bromine (406 mg, 2.54 mmol, 2.5 equiv), chlorine (180 mg, 2.54 mmol, 2.5 equiv) and dichloromethane (0.15 mL) were condensed at −196 °C onto [NPr_4_]Cl (225 mg, 1.01 mmol, 1.0 equiv). Thawing to room temperature and slowly cooling to −20 °C resulted in the formation of crystals of [NPr_4_][Cl(BrCl)_4_]. Raman (1064 nm, 77 K, 150 mW): $\tilde{\nu }$ = 2980(vw), 2955(vw), 2939(vw), 2922(vw), 2897(vw), 1457(vw), 1448(vw), 1316(vw), 385(s, *ν*_s_ Br–Cl), 369(vs., *ν*_as_ Br–Cl), 282(w, *ν*_s_ Br–Cl of [Cl(BrCl)]^−^), 170(vw, sh), 153(w, sh), 142(m) cm^−1^.

**[NEt_4_][Cl(BrCl)_5_]**: Bromine (834 mg, 5.22 mmol, 5.0 equiv), chlorine (370 mg, 5.22 mmol, 5.0 equiv) and dichloromethane (0.15 mL) were condensed at −196 °C onto [NEt_4_]Cl (173 mg, 1.04 mmol, 1.0 equiv). Thawing to room temperature and slowly cooling to −40 °C resulted in the formation of crystals of [NEt_4_][Cl(BrCl)_5_]. Raman (1064 nm, 77 K, 150 mW): $\tilde{\nu }$ = 2987(vw), 2949(vw), 2937(vw), 1456(vw), 1295(vw), 1117(vw), 673(vw), 506(vw), 399(s, *ν*_s_ Br–Cl), 383(vs., *ν*_as_ Br–Cl), 290(vs., *ν* Br–Br), 154(vw), 138(vw) cm^−1^.

**[PNP][Cl(BrCl)_6_]**: Bromine (618 mg, 3.87 mmol, 7.5 equiv), chlorine (274 mg, 3.87 mmol, 7.5 equiv) and dichloromethane (0.15 mL) were condensed at −196 °C onto [PNP]Cl (296 mg, 0.52 mmol, 1.0 equiv). Thawing to room temperature and slowly cooling to −40 °C resulted in the formation of crystals of [PNP][Cl(BrCl)_6_]. Raman (1064 nm, 77 K, 150 mW): $\tilde{\nu }$ = 3062(w), 2987(vw), 1590(vw), 1112(vw), 1028(vw) 1001(w) 702(vw), 668(vw), 617(vw), 406(m, *ν*_s_ Br–Cl), 390(vs., *ν*_as_ Br–Cl), 296(vw), 269(vw) cm^−1^.

**Standard procedure of the interhalogenation**: The substrate (0.32 mmol, 1.0 equiv) was dissolved in dry DCM (3.2 mL) and cooled to −78 °C. The ionic liquid [NEt_3_Me][Cl(BrCl)_2_] was added (60.4 mg, 0.16 mmol, 0.50 equiv) and the reaction mixture was stirred at −78 °C until thin layer chromatography showed conversion of the substrate (alkenes: <1 min; alkyne and α,β‐unsaturated compounds: 30–60 min). Thereafter water (3.0 mL) was added and the reaction mixture was warmed to RT. The aqueous phase was extracted with DCM (3×5.0 mL), the combined organic phases were dried over MgSO_4_ and the solvent was removed under reduced pressure. The crude product was purified by column chromatography (SiO_2_, *n*‐pentane/EtOAc). For a detailed assignment of the ^1^H and ^13^C NMR spectroscopic shifts see Supporting Information (Table S4, Figures S13–S30).

**6‐Bromo‐7‐chloro‐2,2,3,3,10,10,11,11‐octamethyl‐4,9‐dioxa‐3,10‐disiladodecane (A)**: ^1^H NMR (500 MHz, RT, CDCl_3_): *δ*=4.48 (ddd, ^3^ *J*=9.2, 5.7, 1.9 Hz, 1 H), 4.26 (ddd, ^3^ *J*=8.7, 5.8, 1.9 Hz, 1 H), 3.98–3.83 (m, 2 H), 3.88–3.76 (m, 2 H), 0.90 (s, 18 H), 0.10–0.09 (m, 12 H); see Figure S13. ^13^C{^1^H} NMR (126 MHz, RT, CDCl_3_): *δ*=65.2, 64.3, 59.4, 53.9, 25.9 (6×C), 18.4 (2×C), −5.2 (4×C); see Figure S14. ATR‐IR: $\tilde{\nu }$ = 2954, 2930, 2858, 1471, 1257, 1096, 839, 777 cm^−1^. HR‐MS (ESI+): *m*/*z* calcd for C_16_H_37_BrClO_2_Si_2_+H^+^: 431.1199 [*M*+H]^+^; found: 431.1192.

**(2‐Bromo‐3‐chloro‐3‐methylbutoxy)triisopropylsilane (B)**: ^1^H NMR (500 MHz, RT, CDCl_3_): *δ*=4.36 (dd, *J=*11.2, 3.4 Hz, 1 H), 4.18 (dd, *J=*6.9, 3.5 Hz, 1 H), 4.07 (dd, *J=*11.1, 6.8 Hz, 1 H), 1.80 (s, 3 H), 1.70 (s, 3 H), 1.12–1.07 (m, 21 H).; see Figure S15. ^13^C{^1^H} NMR (126 MHz, RT, CDCl_3_): *δ*=70.6, 66.1, 65.7, 33.2, 29.0, 18.1, 12.1; see Figure S16. ATR‐IR: $\tilde{\nu }$ = 2942, 2867, 2190, 1461, 1387, 1123, 1104, 882, 773 cm^−1^. HR‐MS (ESI+): *m*/*z* calcd for C_14_H_30_BrClOSi+Na^+^: 379.0830 [*M*+Na]^+^; found: 379.0829.

**(4‐Bromo‐3‐chloro‐3‐methylbutoxy)triisopropylsilane (C)**: ^1^H NMR (500 MHz, RT, CDCl_3_): *δ*=3.99–3.88 (m, 2 H), 3.78 (d, *J=*10.3 Hz, 1 H), 3.67 (d, *J=*10.3 Hz, 1 H), 2.20–2.12 (m, 2 H), 1.72 (s, 3 H), 1.13–1.02 (m, 21 H); see Figure S17. ^13^C{^1^H} NMR (126 MHz, RT, CDCl_3_): *δ*=70.2, 60.2, 43.7, 43.0, 29.8, 18.2, 12.1; see Figure S18. ATR‐IR: $\tilde{\nu }$ =2943, 2891, 2867, 1737, 1463, 1379, 1217, 1105, 882, 738 cm^−1^. HR‐MS (ESI+): *m*/*z* calcd for C_14_H_30_BrClOSi+Na^+^: 379.0830 [*M*+Na]^+^; found: 379.0840.

**(2‐Bromo/*chloro*‐3‐chloro/*bromo*propoxy)triisopropylsilane, regioisomeric ratio=1:1 (D)**: ^1^H NMR (500 MHz, RT, CDCl_3_): *δ*=4.18–4.13 (m, 1 H), 4.12–4.05 (m, 3 H), 4.01–3.96 (m, 2 H), 3.93 (q, *J=*4.9 Hz, 1 H), 3.88 (dd, *J=*11.2, 4.9 Hz, 1 H), 3.80 (dd, *J=*10.5, 6.8 Hz, 1 H), 3.67 (dd, *J=*10.5, 4.8 Hz, 1 H), 1.15–1.00 (m, 42 H); see Figure S19. ^13^C{^1^H} NMR (126 MHz, RT, CDCl_3_): *δ*=64.8, 64.1, 60.2, 52.3, 45.0, 33.4, 18.1 (2×C), 12.1 (2×C); see Figure S20. ATR‐IR: $\tilde{\nu }$ = 2944, 2892, 2867, 1737, 1461, 1380, 1143, 1110, 1069, 1000, 881, 792 cm^−1^. HR‐MS (ESI+): *m*/*z* calcd for C_12_H_26_BrClOSi+Na^+^: 351.0517 [*M*+Na]^+^; found: 351.0520.

**2‐Bromo‐3‐chloro‐3‐methylbutyl‐3,5‐dinitrobenzoate (E)**: ^1^H NMR (500 MHz, RT, CDCl_3_): *δ*=9.24 (t, *J=*2.1 Hz, 1 H), 9.18 (d, *J=*2.1 Hz, 2 H), 5.13 (dd, *J=*12.1, 3.2 Hz, 1 H), 4.81 (dd, *J=*12.1, 8.8 Hz, 1 H), 4.51 (dd, *J=*8.8, 3.1 Hz, 1 H), 1.87 (s, 3 H), 1.78 (s, 3 H); see Figure S21. ^13^C{^1^H} NMR (126 MHz, RT, CDCl_3_): *δ*=162.2, 148.9, 133.4, 129.7, 122.8, 69.0, 68.1, 59.3, 33.2, 28.1; see Figure S22. ATR‐IR: $\tilde{\nu }$ = 3098, 2955, 2925, 1735, 1542, 1343, 1274, 1159, 921, 719 cm^−1^. HR‐MS (ESI+): *m*/*z* calcd for C_12_H_12_BrClN_2_O_6_+Na^+^: 416.9459 [*M*+Na]^+^; found: 416.9463.

**3‐Bromo‐4‐chloro‐4‐phenylbutan‐2‐one (F)**: ^1^H NMR (500 MHz, RT, CDCl_3_): *δ*=7.44–7.37 (m, 5 H), 5.25 (d, *J=*11.2 Hz, 1 H), 4.69 (d, *J=*11.2 Hz, 1 H), 2.48 (s, 3 H); see Figure S23. ^13^C{^1^H} NMR (126 MHz, RT, CDCl_3_): *δ*=198.7, 137.4, 129.6, 128.9, 128.1, 59.9, 53.7, 27.0; see Figure S24. ATR‐IR: $\tilde{\nu }$ = 3066, 3032, 2970, 1722, 1455, 1362, 1218, 733 cm^−1^. HR‐MS (ESI+): *m*/*z* calcd for C_10_H_10_BrClO+Na^+^: 282.9496 [*M*+Na]^+^; found: 282.9489; *m*/*z* calcd for C_10_H_10_BrClO+K^+^: 298.9236 [*M*+K]^+^; found: 289.9227.

**Ethyl‐2‐bromo‐3‐chloro‐3‐phenylpropanoate (G)**: ^1^H NMR (500 MHz, RT, CDCl_3_): *δ*=7.43–7.36 (m, 5 H), 5.27 (d, *J=*11.3 Hz, 1 H), 4.62 (d, *J=*11.3 Hz, 1 H), 4.36 (q, *J=*7.2 Hz, 2 H), 1.37 (t, *J=*7.1 Hz, 3 H); see Figure S25. ^13^C{^1^H} NMR (126 MHz, RT, CDCl_3_): *δ*=167.8, 137.2, 129.6, 128.9, 128.1, 62.7, 61.0, 47.7, 14.0; see Figure S26. ATR‐IR: $\tilde{\nu }$ = 3034, 2982, 2936, 1739, 1455, 1266, 1143, 1017, 869, 772 cm^−1^. HR‐MS (ESI+): *m*/*z* calcd for C_11_H_12_BrClO_2_+Na^+^: 312.9601 [*M*+Na]^+^; found: 312.9604.

**2‐Bromo‐3‐chloro‐1,3‐diphenylpropan‐1‐one (H)**: ^1^H NMR (500 MHz, RT, CDCl_3_): *δ*=8.12–8.10 (m, 2 H), 7.68–7.65 (m, 1 H), 7.57–7.53 (m, 4 H), 7.46–7.40 (m, 3 H), 5.61 (s, 2 H); see Figure S27. ^13^C{^1^H} NMR (126 MHz, RT, CDCl_3_): *δ*=191.3, 137.9, 134.6, 134.3, 129.5, 129.1, 129.0, 128.9, 128.3, 59.9, 47.6; see Figure S28. ATR‐IR: $\tilde{\nu }$ = 3063, 3033, 1685, 1448, 1269, 1229, 976, 776, 731 cm^−1^. HR‐MS (ESI+): *m*/*z* calcd for C_15_H_12_BrClO+Na^+^: 344.9652 [*M*+Na]^+^; found: 344.9656; *m*/*z* calcd for C_15_H_12_BrClO+K^+^: 360.9392 [*M*+K]^+^; found: 360.9394.

**(*E*)‐6‐Bromo‐7‐chloro‐2,2,3,3,10,10,11,11‐octamethyl‐4,9‐dioxa‐3,10‐disiladodec‐6‐ene (I)**: ^1^H NMR (500 MHz, RT, CDCl_3_): *δ*=4.55–4.50 (m, 4 H), 0.92–0.91 (m, 18 H), 0.12–0.10 (m, 12 H); see Figure S29. ^13^C{^1^H} NMR (126 MHz, RT, CDCl_3_): *δ*=130.7, 122.4, 66.0, 64.7, 26.0, 18.5, −5.03; see Figure S30. ATR‐IR: $\tilde{\nu }$ = 2953, 2930, 2886, 2858, 1738, 1471, 1363, 1254, 1111, 836, 777 cm^−1^. HR‐MS (ESI+): *m*/*z* calcd for C_16_H_34_BrClO_2_Si_2_+Na^+^: 451.0861 [*M*+Na]^+^; found: 451.0869; *m*/*z* calcd for C_16_H_34_BrClO_2_Si_2_+K^+^: 467.0601 [*M*+K]^+^; found: 467.0616.

## Conflict of interest

The authors declare no conflict of interest.

## Supporting information

As a service to our authors and readers, this journal provides supporting information supplied by the authors. Such materials are peer reviewed and may be re‐organized for online delivery, but are not copy‐edited or typeset. Technical support issues arising from supporting information (other than missing files) should be addressed to the authors.

Supplementary
