# Supplementary material for: In Situ Synthesis and Applications for Polyinterhalides Based on BrCl
Source: Chemistry. 2020 Oct 22;26(66):15183–9. doi: 10.1002/chem.202001267 (PMC7814667; doi:10.1002/chem.202001267)
Supplement: Supplementary file 1 — Supplementary [file CHEM-26-15183-s001.pdf]

Chemistry–A European Journal

Supporting Information

In Situ Synthesis and Applications for Polyinterhalides Based on BrCl

Benjamin Schmidt,[a] Sebastian Ponath,[b] Johannes Hannemann,[a] Patrick Voßnacker,[a] Karsten Sonnenberg,[a] Mathias Christmann,[b] and Sebastian Riedel*[a]

Supporting Information

Table of Content

1. **Results and Discussion** 2
   a. **Crystal Data** 2
   b. **Solid State Structures** 4
   c. **Raman Spectra** 6
   d. **IR Spectra** 9
   e. **Calculated Thermochemistry** 9
   f. **Interhalogenation: Substrate Scope** 10
   g. **NMR Spectra** 11
   h. **Crystal Packing / Intermolecular interactions** 20
   i. **Long Term Stability Studies** 22
   j. **Computed Vibrational Frequencies** 24
   k. **Additional Information** 28
   l. **xyz-Files of the Calculated Molecules** 31

1. Results and Discussion

a. Crystal Data

Table S1. Crystal data of the synthesized compounds.

Empirical formula	$C_8H_{20}BrCl_2N$ (**1**)	$C_{16}H_{40}Br_3Cl_5N_2$ (**2**)	$C_8H_{20}Br_3Cl_4N$ (**3**)
Formula weight	281.06	677.48	511.78
Temperature/K	100.0	100.0	100.0
Crystal system	orthorhombic	monoclinic	monoclinic
Space group	*Pnma*	$P2_1/m$	$P2_1/c$
a/Å	11.9218(16)	7.7583(4)	12.0185(6)
b/Å	12.4514(15)	12.8611(7)	10.1733(5)
c/Å	17.4209(17)	14.2896(8)	14.5527(6)
α/°	90	90	90
β/°	90	103.750(2)	99.093(2)
γ/°	90	90	90
Volume/Å^3	2586.0(5)	1384.96(13)	1756.97(14)
Z	8	2	4
ρ_{calc}g/cm^3	1.444	1.625	1.935
μ/mm^{-1}	3.551	4.858	7.476
F(000)	1152.0	680.0	992.0
Crystal size/mm^3	0.3 × 0.2 × 0.11	0.458 × 0.453 × 0.28	0.58 × 0.31 × 0.24
Radiation	MoKα (λ = 0.71073)	MoKα (λ = 0.71073)	MoKα (λ = 0.71073)
2Θ range for data collection/°	4.676 to 56.61	5.406 to 56.634	4.906 to 56.68
Reflections collected	24805	65037	30162
Independent reflections	3364 [R_{int} = 0.0595, R_{sigma} = 0.0344]	3601 [R_{int} = 0.0524, R_{sigma} = 0.0176]	4370 [R_{int} = 0.0929, R_{sigma} = 0.0545]
Data/restraints/parameters	3364/0/122	3601/0/134	4370/0/149
Goodness-of-fit on F^2	1.054	1.048	1.079
Final R indexes [I>=2σ (I)]	R_1 = 0.0277, wR_2 = 0.0537	R_1 = 0.0200, wR_2 = 0.0434	R_1 = 0.0426, wR_2 = 0.1008
Final R indexes [all data]	R_1 = 0.0404, wR_2 = 0.0571	R_1 = 0.0259, wR_2 = 0.0456	R_1 = 0.0609, wR_2 = 0.1096
Largest diff. peak/hole / e Å^{-3}	0.39/-0.55	0.37/-0.52	0.87/-1.56
CCDC deposition numbers	1965315	1984581	1965317

Table S2. Crystal data of the synthesized compounds.

Empirical formula	$C_{12}H_{28}Br_4Cl_5N$ (**4**)	$C_8H_{20}Br_{5.6}Cl_{5.4}N$ (**5**)	$C_8H_{20}Br_{2.32}Cl_{2.68}N$	$C_{15}H_{12}BrClO$
Formula weight	683.24	769.17	410.47	323.61
Temperature/K	100.0	100.0	100.0	100.0
Crystal system	tetragonal	monoclinic	monoclinic	monoclinic
Space group	$I\bar{4}$	$P2_1/n$	$P2_1/n$	*Cc*
a/Å	11.8884(13)	14.6745(13)	8.4748(3)	5.7073(3)
b/Å	11.8884(13)	10.3054(8)	13.9985(5)	25.3888(13)
c/Å	8.5997(9)	16.5643(14)	12.9242(4)	9.0603(5)
α/°	90	90	90	90
β/°	90	114.001(3)	93.8220(10)	95.499(2)
γ/°	90	90	90	90
Volume/Å^3	1215.4(3)	2288.4(3)	1529.85(9)	1306.81(12)
Z	2	4	4	4
ρ_{calc}g/cm^3	1.867	2.233	17.820	1.645
μ/mm^{-1}	7.169	10.450	6.564	6.030
F(000)	664.0	1451.0	806.2	648.0
Crystal size/mm^3	0.26 × 0.21 × 0.21	0.403 × 0.339 × 0.298	0.319 × 0.303 × 0.206	0.322 × 0.075 × 0.033
Radiation	MoKα (λ = 0.71073)	MoKα (λ = 0.71073)	Mo Kα (λ = 0.71073)	CuKα (λ = 1.54178)
2Θ range for data collection/°	4.846 to 56.49	3.134 to 56.67	4.3 to 66.34	6.964 to 139.3
Reflections collected	9392	54685	38582	16304
Independent reflections	1516 [R_{int} = 0.0532, R_{sigma} = 0.0375]	5702 [R_{int} = 0.0893, R_{sigma} = 0.0448]	5755 [R_{int} = 0.0363, R_{sigma} = 0.0245]	2313 [R_{int} = 0.0538, R_{sigma} = 0.0345]
Data/restraints/parameters	1516/0/52	5702/7/195	5755/0/151	2313/2/163
Goodness-of-fit on F^2	1.098	1.047	1.059	1.080
Final R indexes [I>=2σ (I)]	R_1 = 0.0255, wR_2 = 0.0570	R_1 = 0.0333, wR_2 = 0.0794	R_1 = 0.0226, wR_2 = 0.0478	R_1 = 0.0343, wR_2 = 0.0832
Final R indexes [all data]	R_1 = 0.0286, wR_2 = 0.0580	R_1 = 0.0475, wR_2 = 0.0857	R_1 = 0.0354, wR_2 = 0.0547	R_1 = 0.0360, wR_2 = 0.0845
Largest diff. peak/hole / e Å^{-3}	0.53/-0.51	0.76/-1.07	0.60/-0.96	0.79/-0.82
CCDC deposition numbers	1965314	1965318	1971174	1984909

b. Solid State Structures

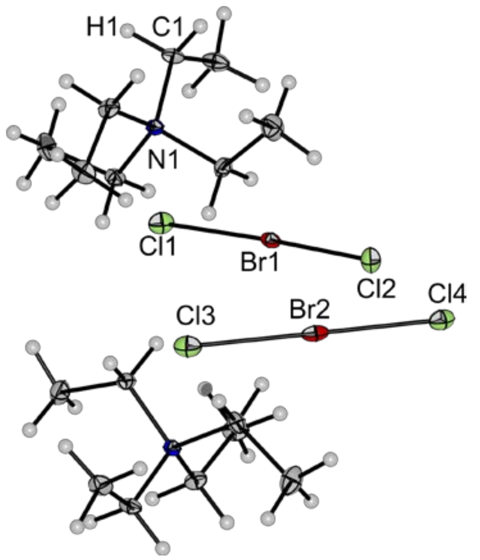

Figure S1. Molecular structure of $[NEt_4][Cl(BrCl)]$ in the solid state with thermal ellipsoids set at 50 % probability. Selected bond lengths [pm] and angles [°]: Cl1-Br1 241.8(1), Cl2-Br1 235.7(1), Cl3-Br2 240.4(1), Cl4-Br2 239.1(1); Cl1-Br1-Cl2 174.8(1), Cl3-Br2-Cl4 179.1(1).

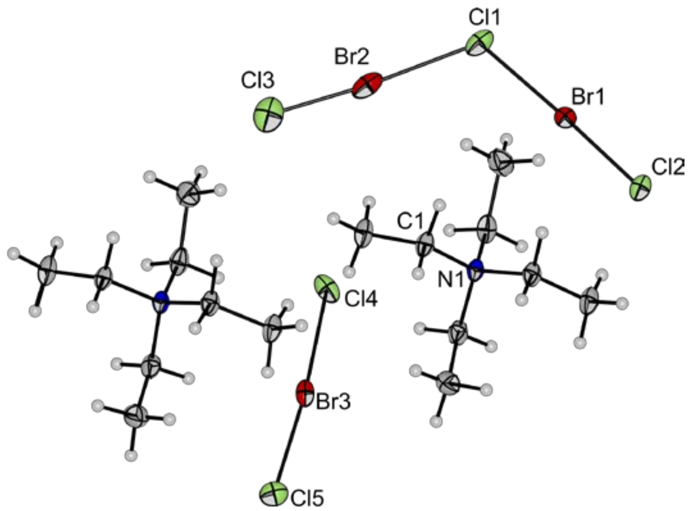

Figure S2. Molecular structure of $[NEt_4]_2[Cl(BrCl)_2][ClBrCl]$ in the solid state with thermal ellipsoids set at 50 % probability. Selected bond lengths [pm] and angles [°]: Cl1-Br1 251.7(1), Cl1-Br2 262.3(1), Br1-Cl2 227.8(1), Br2-Cl3 224.1(1), Cl4-Br3 239.8(1), Cl5-Br3 236.8(1); Cl1-Br1-Cl2 178.0(1), Cl1-Br2-Cl3 175.7(1), Br2-Cl1-Br1 116.6(1), Cl4-Br3-Cl5 174.7(1).

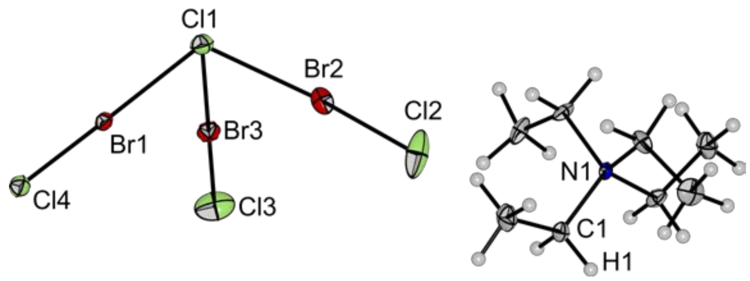

Figure S3. Molecular structure of [NEt$_4$][Cl(BrCl)$_3$] in the solid state with thermal ellipsoids set at 50 % probability. Selected bond lengths [pm] and angles [°]: Cl1-Br1 266.4(2), Cl1-Br2 273.0(2), Cl1-Br3 269.2(2), Br1-Cl4 223.0(2), Br2-Cl2 220.1(2), Br3-Cl3 221.0(2); Cl1-Br1-Cl4 177.6(1), Cl1-Br3-Cl3 178.0(1), Cl1-Br2-Cl2 175.5(1), Br1-Cl1-Br3 90.5(1), Br3-Cl1-Br2 87.7(1), Br1-Cl1-Br2 104.2(1).

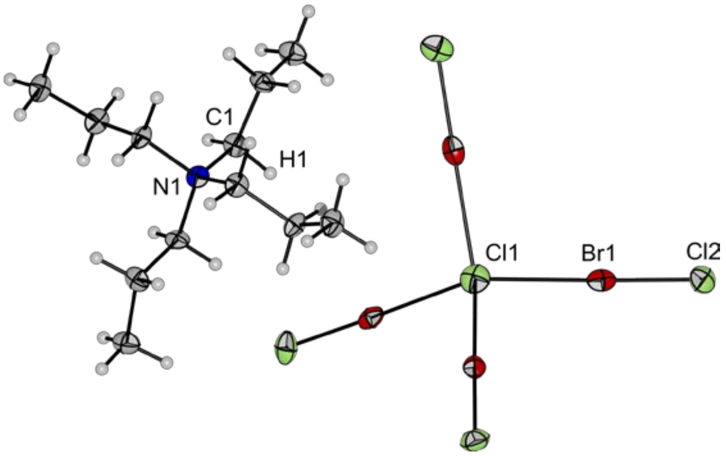

Figure S4. Molecular structure of [NPr$_4$][Cl(BrCl)$_4$] in the solid state with thermal ellipsoids set at 50 % probability. Selected bond lengths [pm] and angles [°]: Cl1-Br1 278.6(1), Br1-Cl2 221.3(2); Cl1-Br1-Cl2 177.7(1), Br1-Cl1-Br1' 100.3(1), 130.1(1).

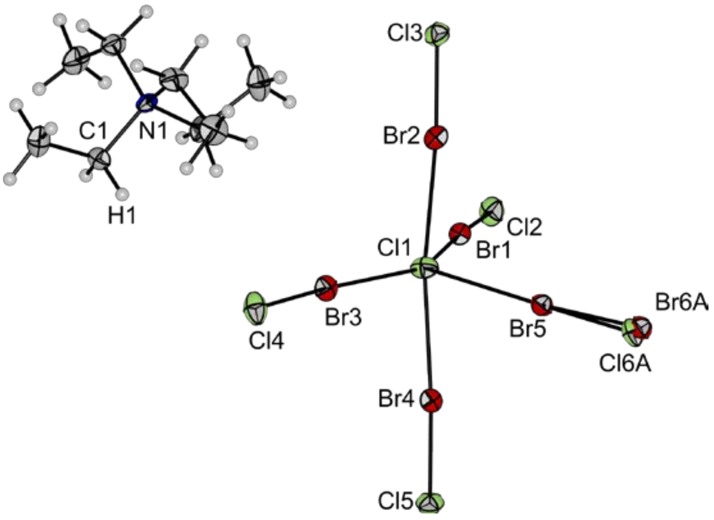

Figure S5. Molecular structure of [NEt$_4$][Cl(BrCl)$_5$] in the solid state with thermal ellipsoids set at 50 % probability. Selected bond lengths [pm] and angles [°]: Cl1-Br1 286.4(2), Cl1-Br2 281.6(2), Cl1-Br3 281.4(2), Cl1-Br4 281.1(2), Cl1-Br5 298.6(2), Br1-Cl2 218.0(2), Br2-Cl3 219.4(2), Br3-Cl4 219.1(2), Br4-Cl5 218.9(2), Br5-Cl6A 218.4(16), Br5-Br6A 231.4(5); Cl1-Br1-Cl2 176.7(1), Cl1-Br2-Cl3 174.9(1), Cl1-Br3-Cl4 175.3(1), Cl1-Br4-Cl5 176.1(1), Cl1-Br5-Cl6A 174.8(8), Br3-Cl1-Br1 148.7(1), Br2-Cl1-Br4 171.5(1);); population of the disorders: Cl6A: 40 %, Br6A: 60 %.

c. Raman Spectra

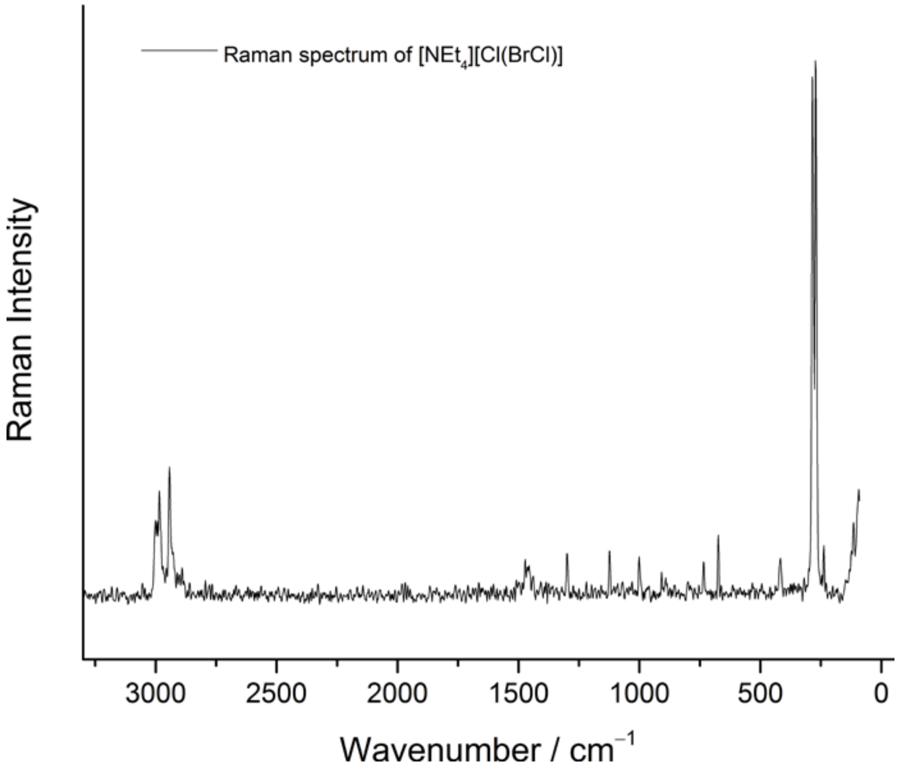

Figure S6. Raman spectrum of a single crystal of [NEt$_4$][Cl(BrCl)], taken at low temperature (–196 °C).

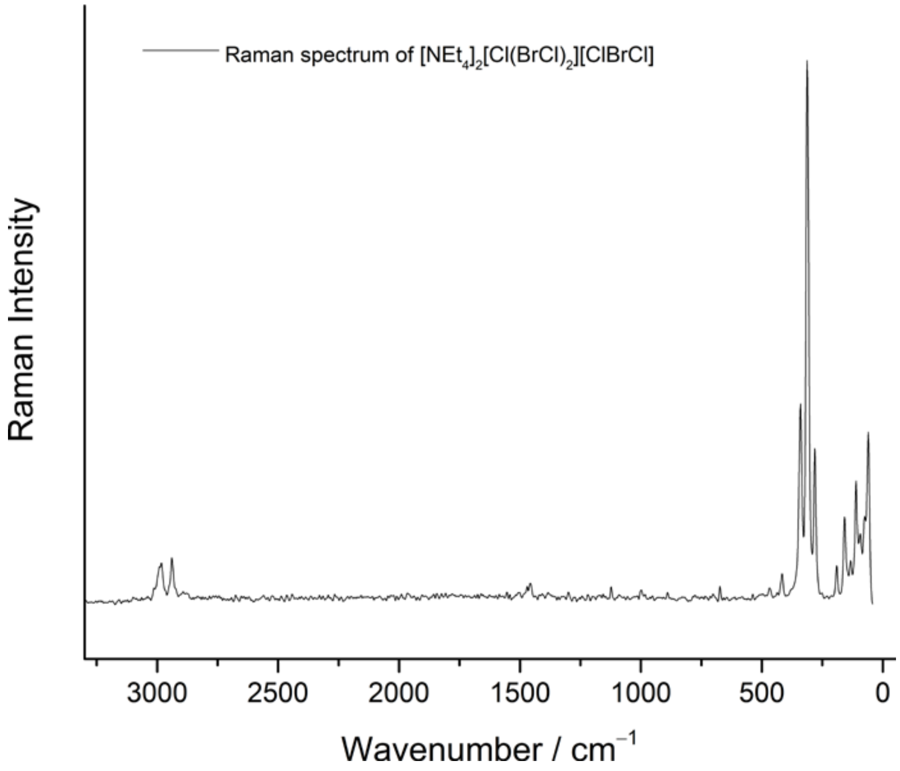

Figure S7. Raman spectrum of a single crystal of [NEt$_4$]$_2$[Cl(BrCl)$_2$][ClBrCl], taken at low temperature (–196 °C).

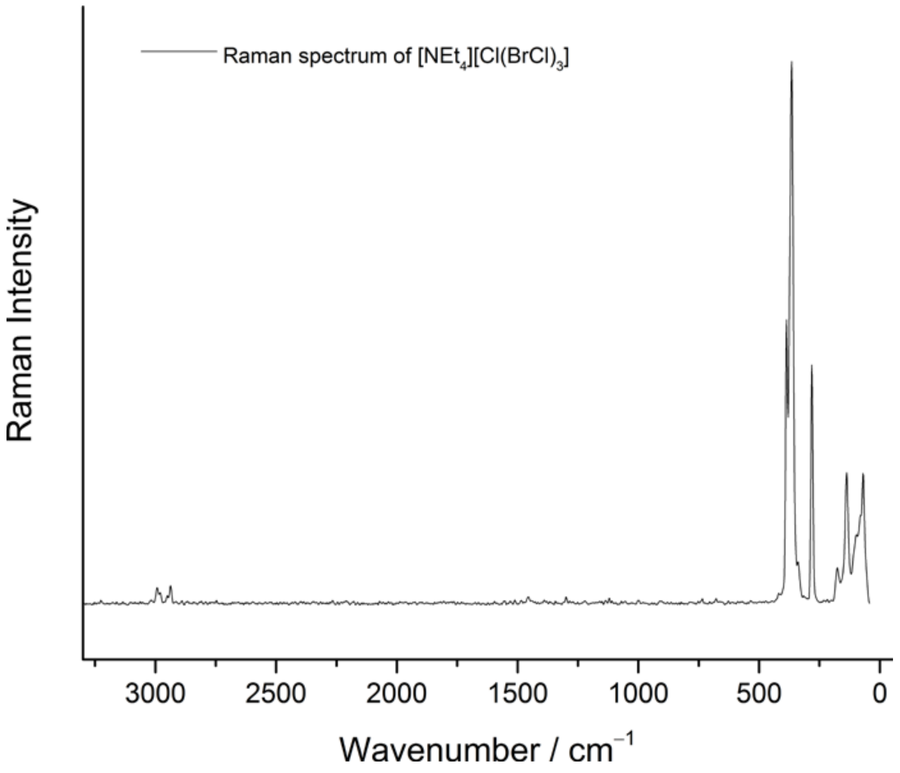

Figure S8. Raman spectrum of a single crystal of $[NEt_4][Cl(BrCl)_3]$, taken at low temperature (–196 °C).

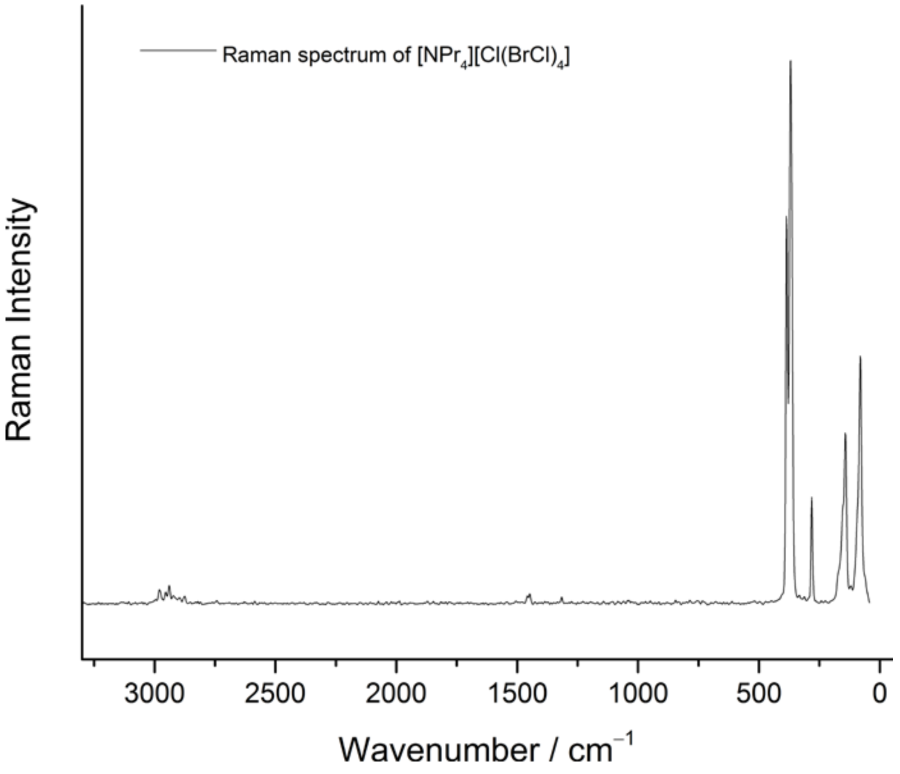

Figure S9. Raman spectrum of a single crystal of $[NPr_4][Cl(BrCl)_4]$, taken at low temperature (–196 °C).

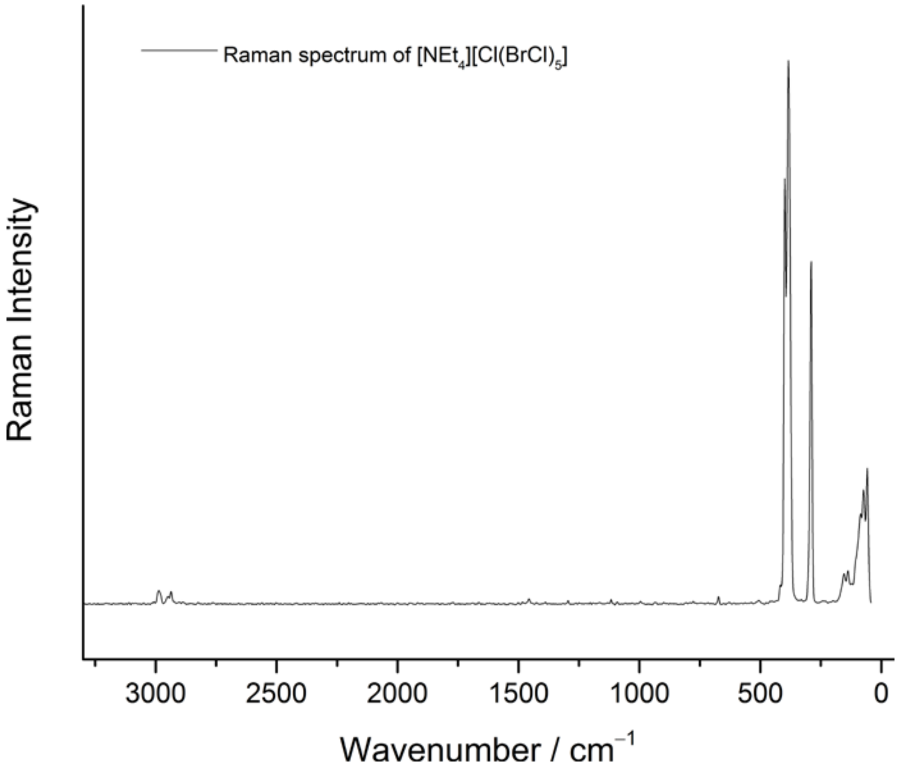

Figure S10. Raman spectrum of a single crystal of $[NEt_4][Cl(BrCl)_5]$, taken at low temperature (–196 °C).

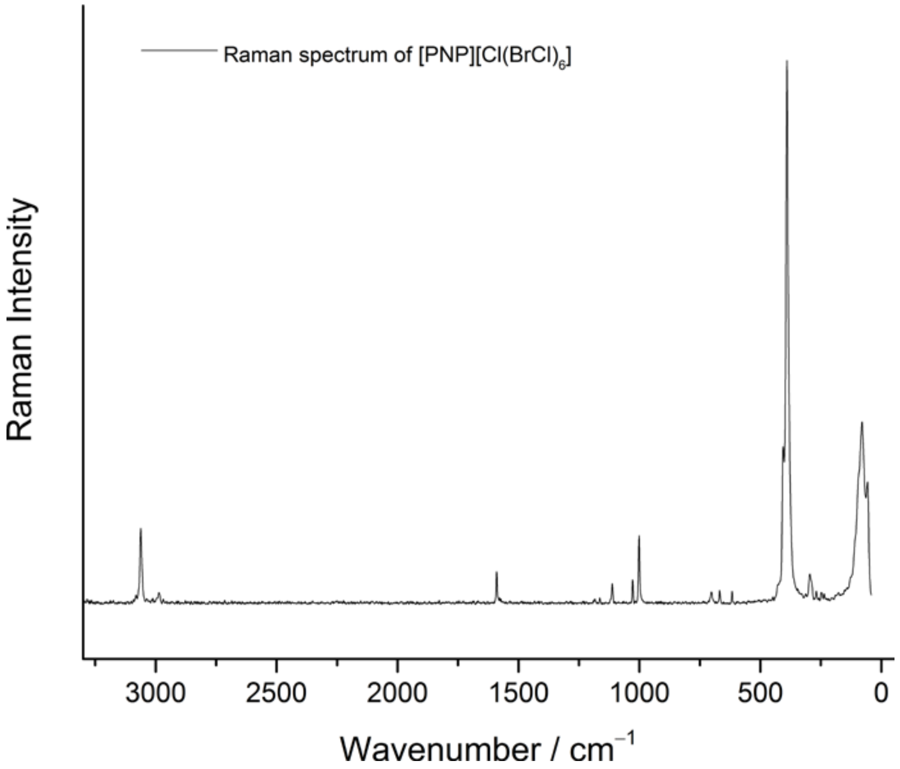

Figure S11. Raman spectrum of a single crystal of $[PNP][Cl(BrCl)_6]$, taken at low temperature (–196 °C).

d. IR Spectra

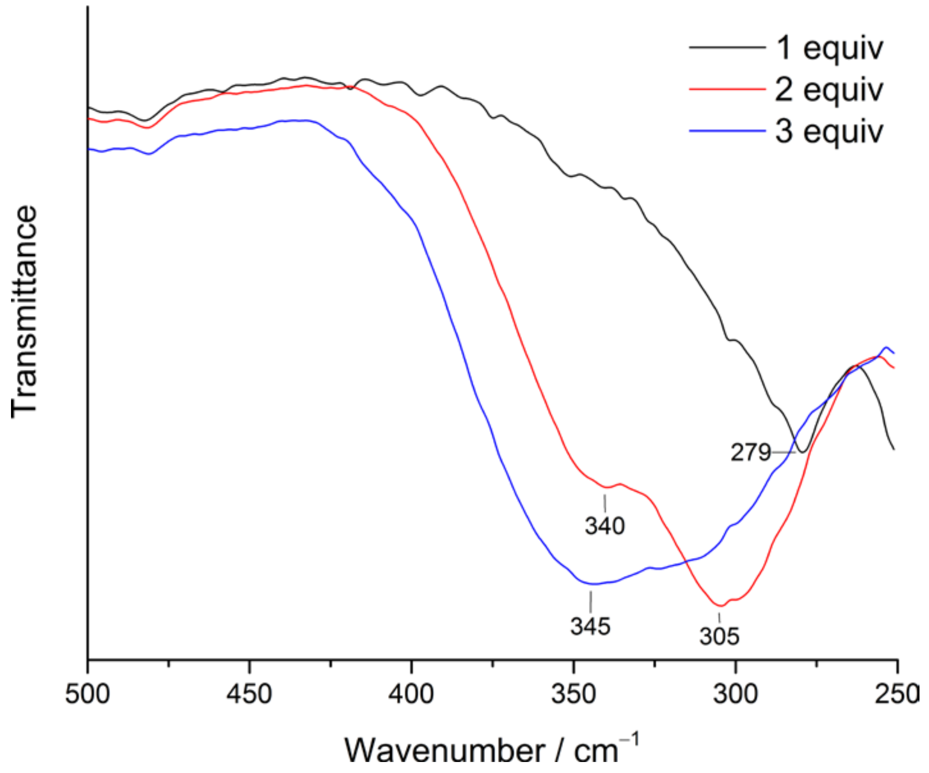

Figure S12. Selected bands of the ATR-IR spectra of RT-ILs obtained from $[N_{2221}]Cl$ and 1 – 3 equiv of BrCl.

e. Calculated Thermochemistry

Table S3. Calculated ΔG (298.15 K, 1 bar) and ΔE values for the formation of $[Cl(BrCl)_n]^-$ calculated at B3LYP-D3(BJ)/def2-TZVPP and SCS-MP2/def2-TZVPP levels.

	B3LYP-D3(BJ)		SCS-MP2	
	**Δ*G* [kJ/mol]**	**Δ*E* [kJ/mol]**	**Δ*G* [kJ/mol]**	**Δ*E* [kJ/mol]**
$[Cl(BrCl)]^-$ + BrCl → $[Cl(BrCl)_2]^-$ (C_{2v})	–48.8	–81.1	–30.4	–62.4
$[Cl(BrCl)_2]^-$ + BrCl → $[Cl(BrCl)_3]^-$ (C_{3v})	–19.0	–54.5	–11.1	–47.5
$[Cl(BrCl)_3]^-$ + BrCl → $[Cl(BrCl)_4]^-$ (T_d)	–9.2	–42.4	–8.1	–39.8
$[Cl(BrCl)_4]^-$ + BrCl → $[Cl(BrCl)_5]^-$ (D_{3h})	7.4	–29.9	12.8	–33.1
$[Cl(BrCl)_5]^-$ + BrCl → $[Cl(BrCl)_6]^-$ (O_h)	8.1	–32.2	9.1	–34.8
2 BrCl → Cl_2 + Br_2	5.2	1.7	5.9	2.4
2 $[Cl(BrCl)_4]^-$ → $[Cl(Cl_2)_4]^-$ + $[Cl(Br_2)_4]^-$	89.6	103.8	114.9	122.0
2 $[Cl(BrCl)_2]^-$ → $[Cl(Cl_2)_2]^-$ + $[Cl(Br_2)_2]^-$	65.9	67.6	78.3	81.4

f. Interhalogenation: Substrate Scope

Table S4. Substrate scope of the interhalogenation with the reactive IL $[NEt_3Me][Cl(BrCl)_2]$.

Nr.	substrate	product	yield
**A**	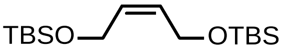	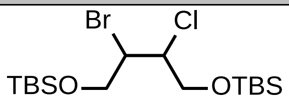	86%
**B**	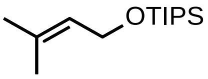	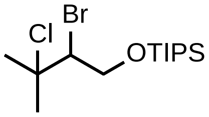	83%
**C**	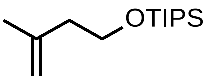	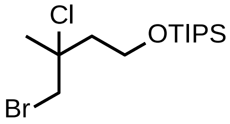	86%
**D**	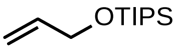	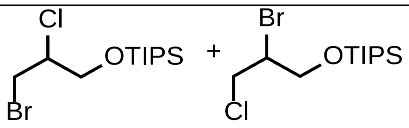 regioisomeric ratio = 1:1	87%
**E**	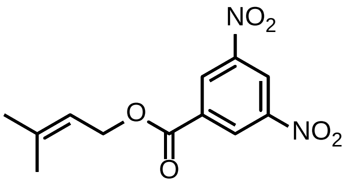	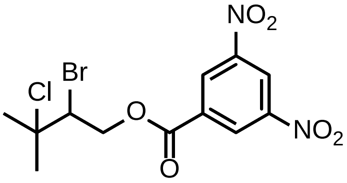	84%
**F**	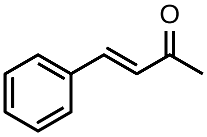	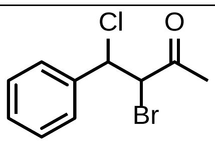	88%
**G**	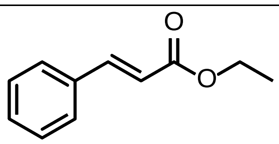	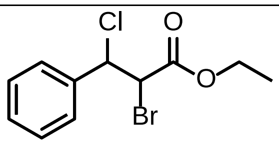	91%
**H**	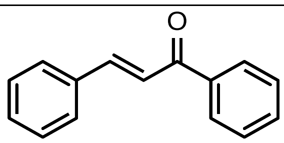	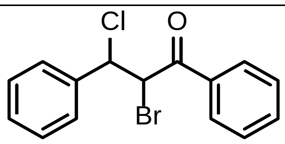	89%
**I**	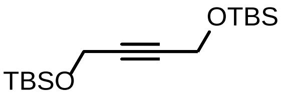	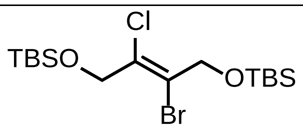	71%

The excellent regio- and diastereoselectivity (*cis*/*trans, E*/*Z*) of all compounds (beside **D**) is in accordance with the classical reactivity of unsaturated substrates with interhalides. The more electrophilic bromine atom forms a bromonium ion which is subsequently attacked by the remaining chloride. The nucleophilic attack on the bromonium ion is directed by the substitution pattern (tertiary>secondary>primary carbocation) of the participating carbon atoms. For Michael-systems the nucleophilic chloride exclusively attacks the benzylic β-position. The solid state structure of compound **H** confirms these considerations.

g. NMR Spectra

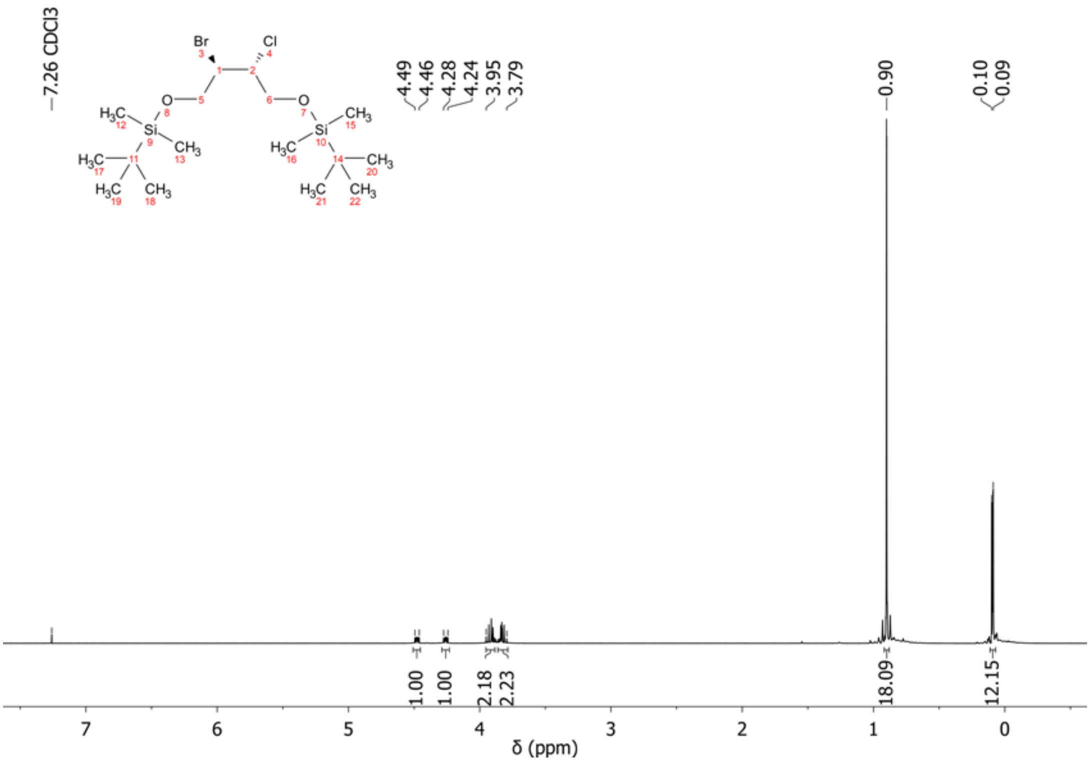

Figure S13. ^{1}H-NMR spectrum of the interhalogenation product **A**.

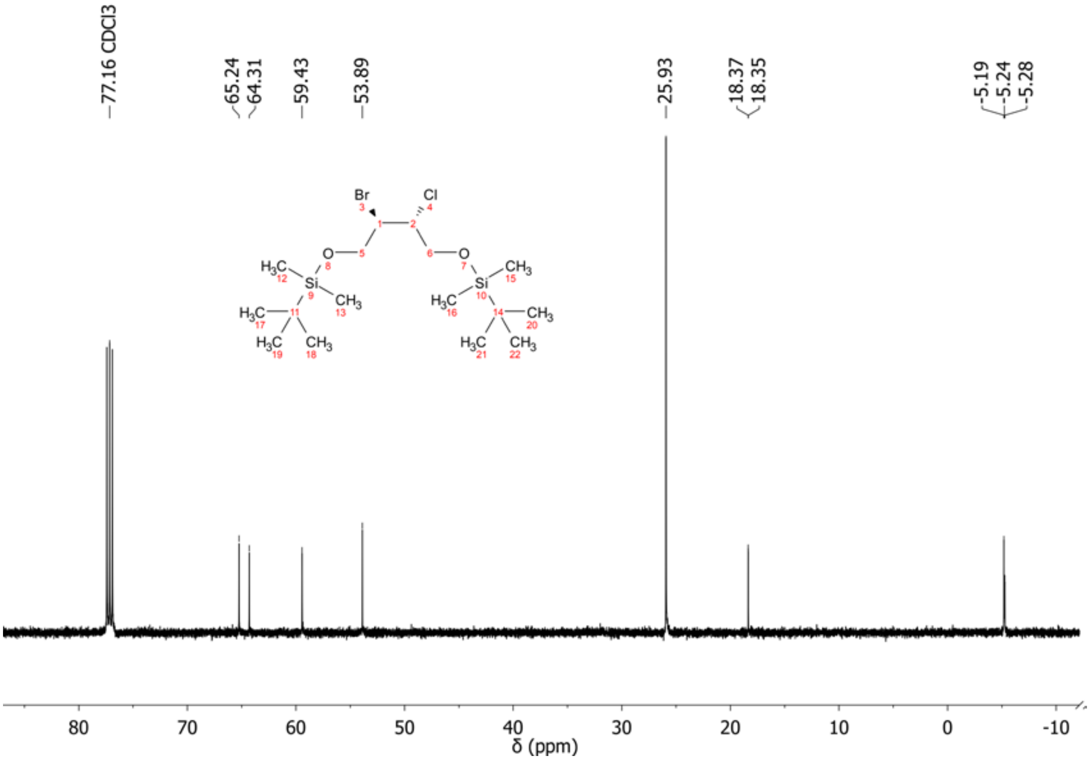

Figure S14. ^{13}C-NMR spectrum of the interhalogenation product **A**.

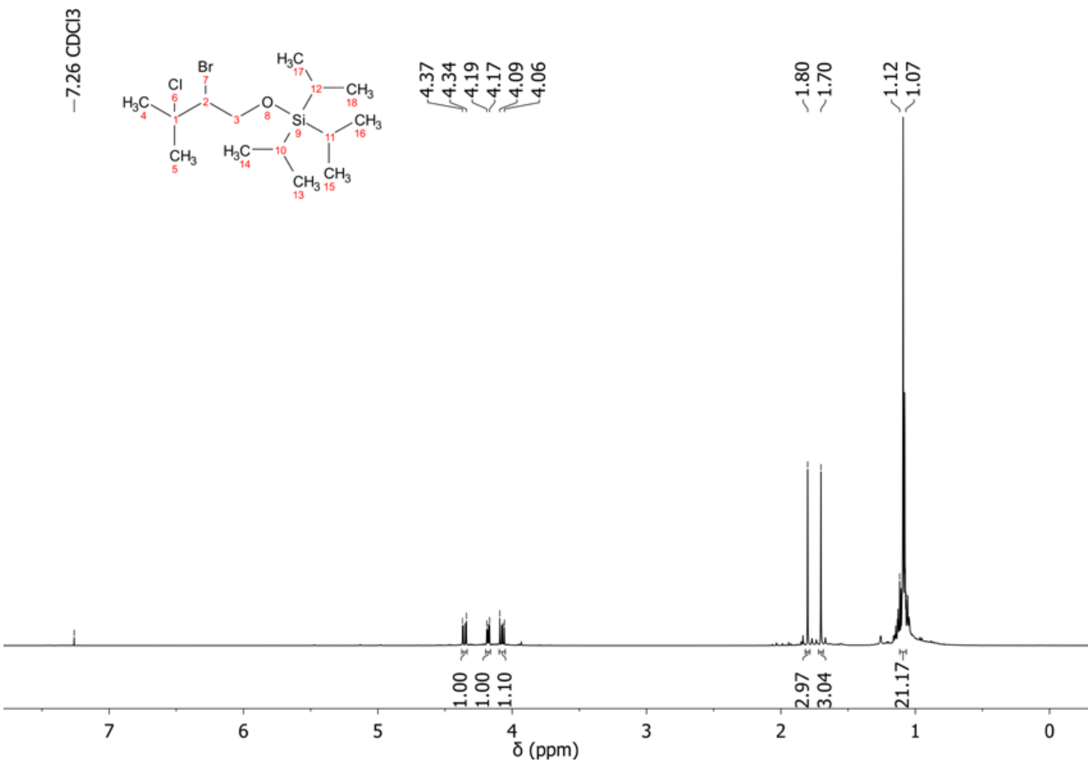

Figure S15. ^{1}H-NMR spectrum of the interhalogenation product **B**.

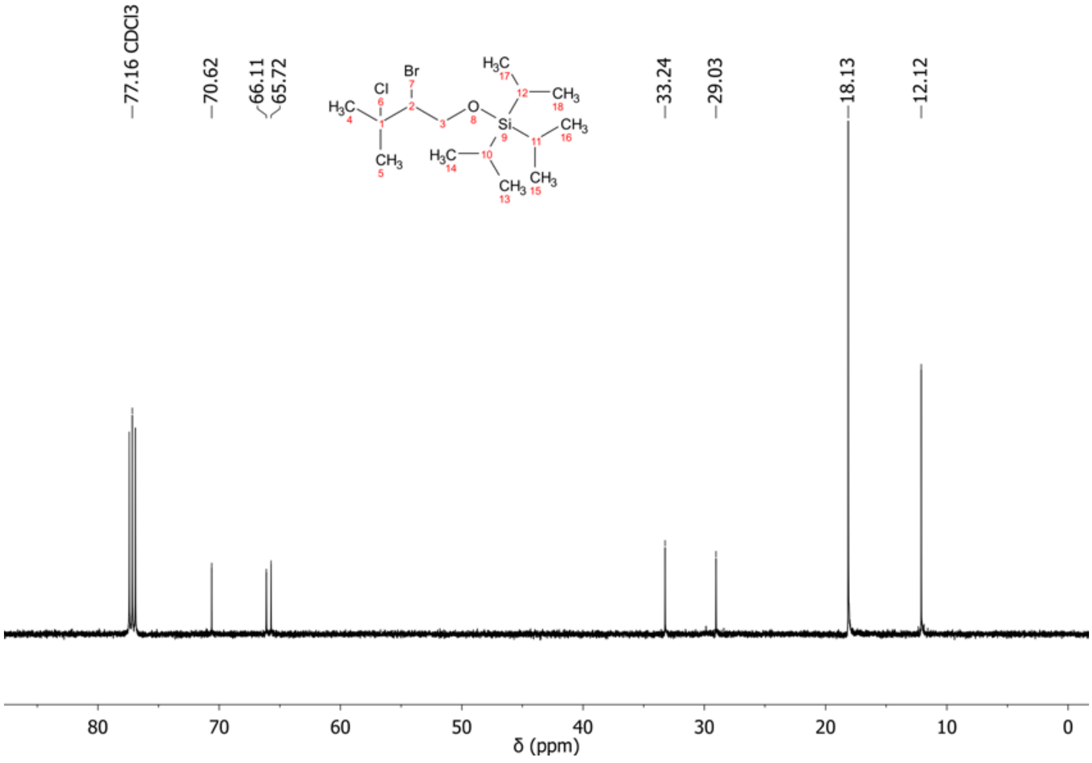

Figure S16. ^{13}C-NMR spectrum of the interhalogenation product **B**.

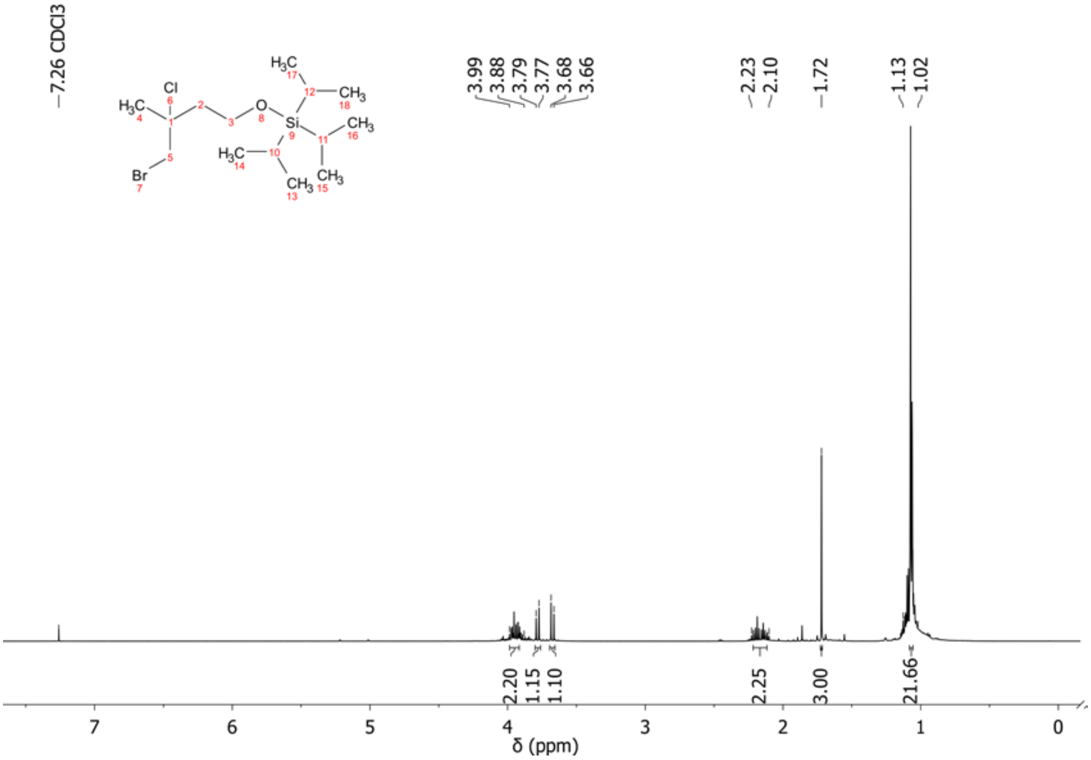

Figure S17. ^{1}H-NMR spectrum of the interhalogenation product **C**.

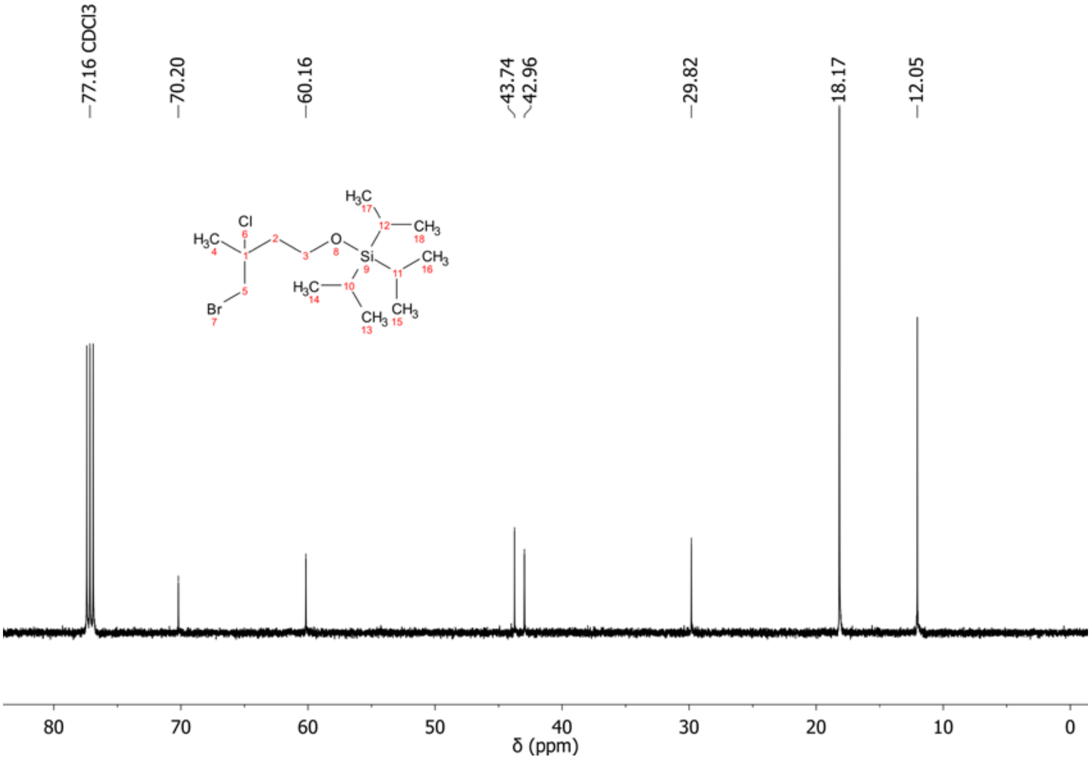

Figure S18. ^{13}C-NMR spectrum of the interhalogenation product **C**.

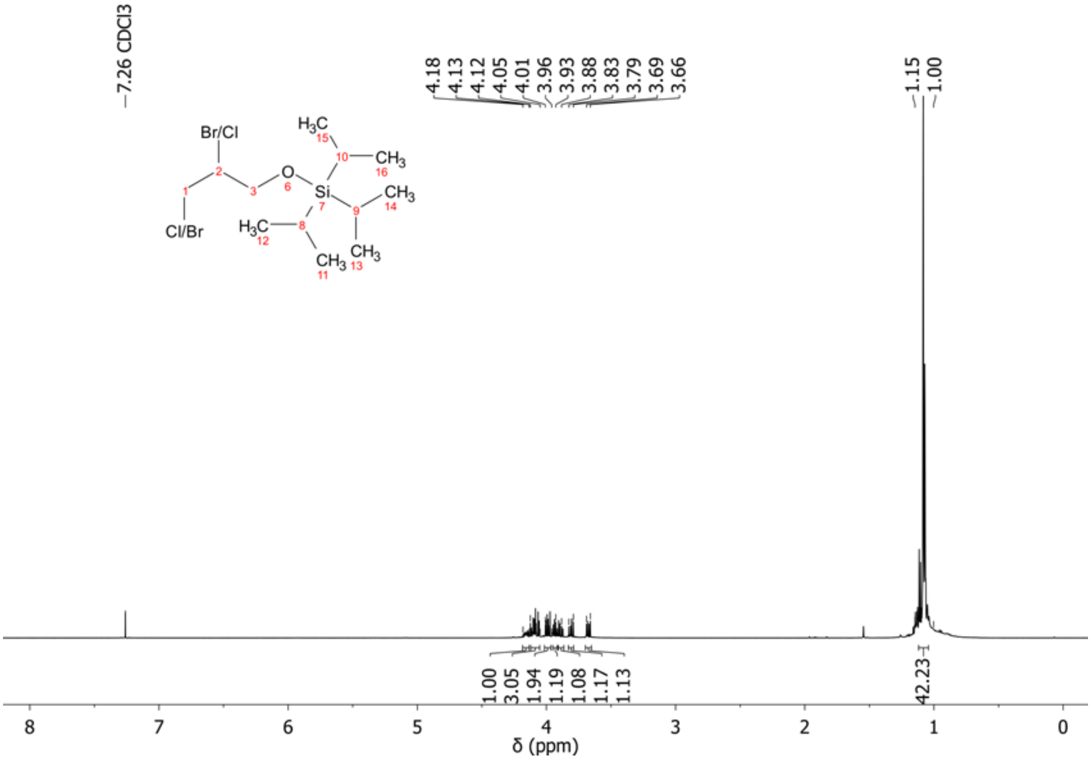

Figure S19. ^{1}H-NMR spectrum of the interhalogenation products **D**.

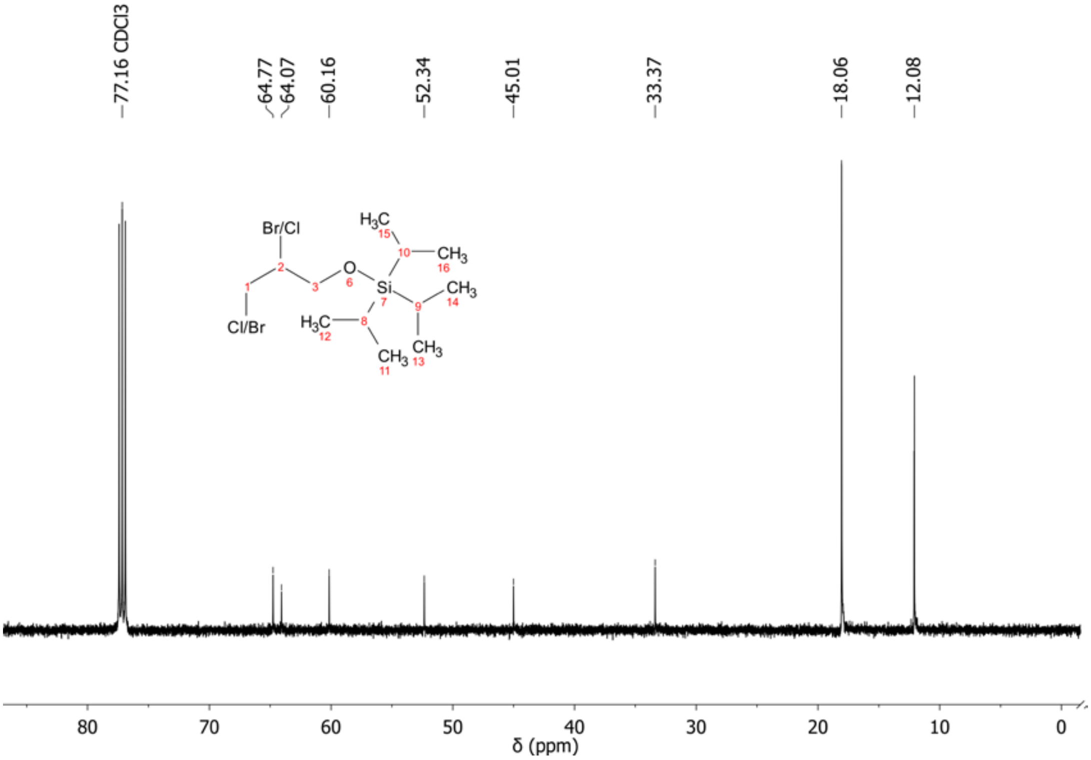

Figure S20. ^{13}C-NMR spectrum of the interhalogenation products **D**.

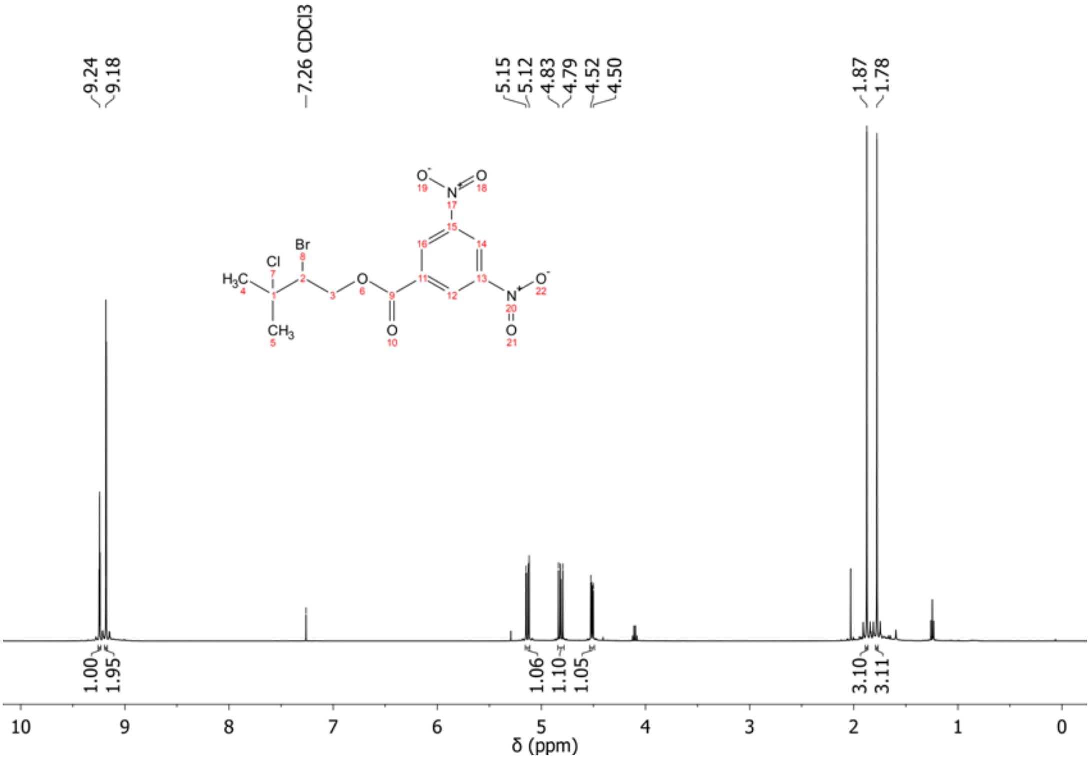

Figure S21. ^{1}H-NMR spectrum of the interhalogenation product **E**.

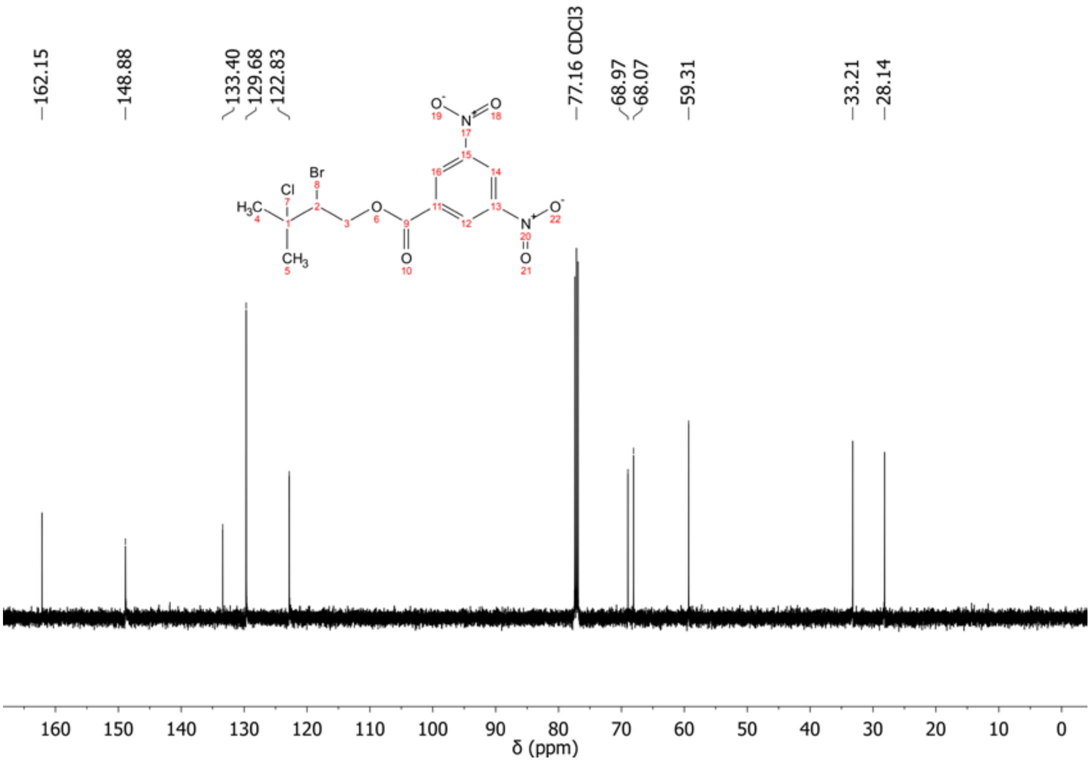

Figure S22. ^{13}C-NMR spectrum of the interhalogenation product **E**.

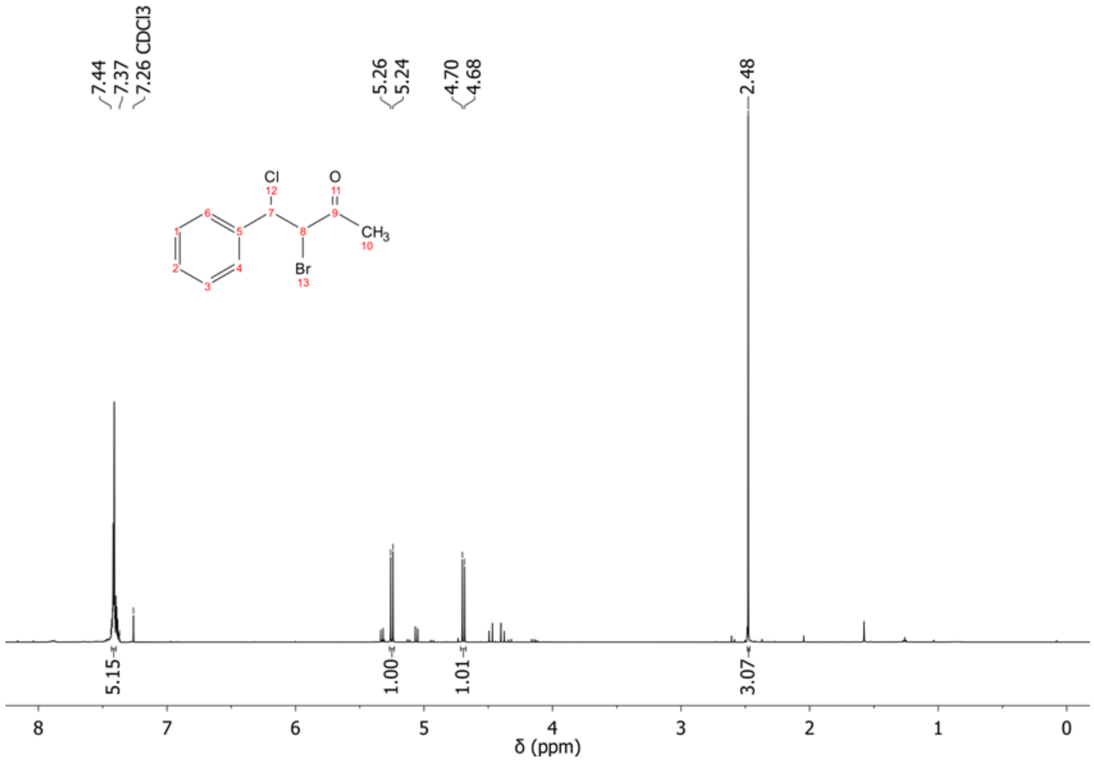

Figure S23. ^{1}H-NMR spectrum of the interhalogenation product **F**.

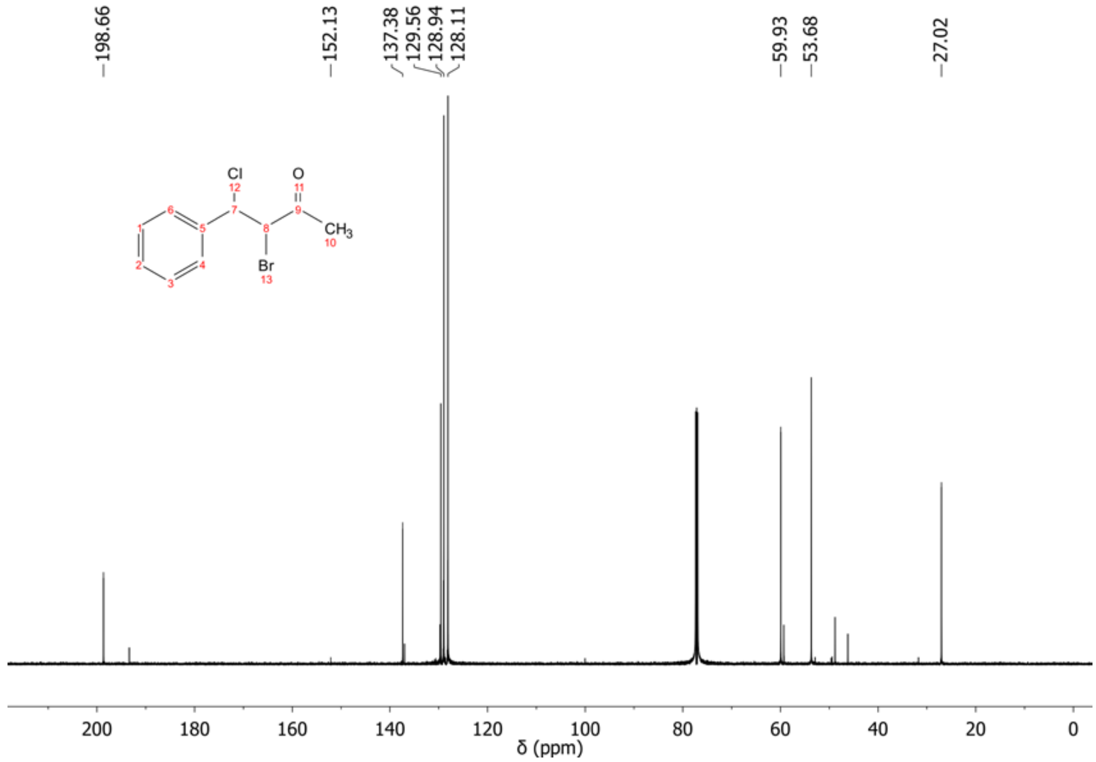

Figure S24. ^{13}C-NMR spectrum of the interhalogenation product **F**.

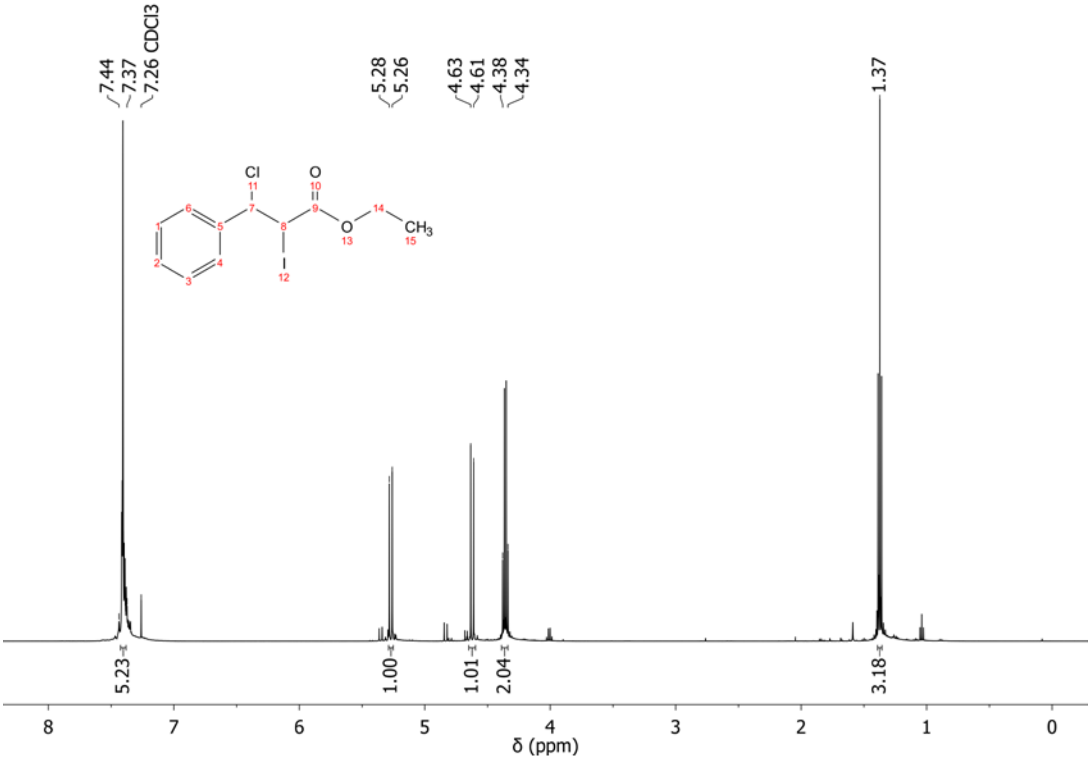

Figure S25. ^{1}H-NMR spectrum of the interhalogenation product **G**.

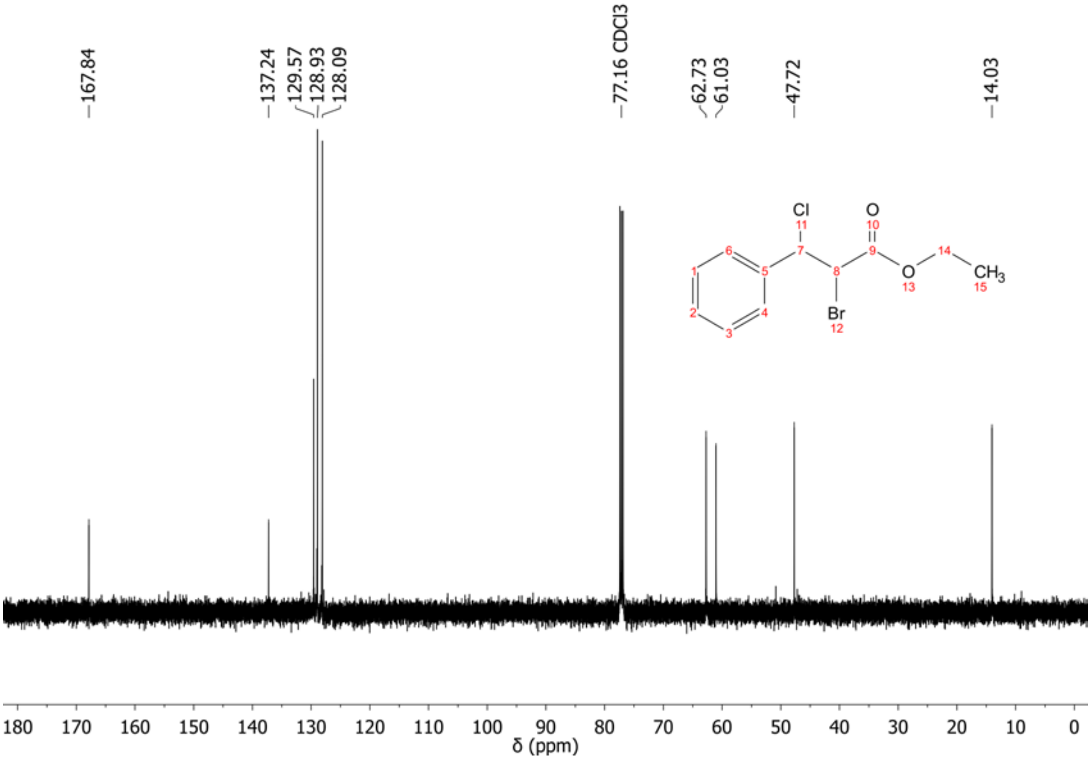

Figure S26. ^{13}C-NMR spectrum of the interhalogenation product **G**.

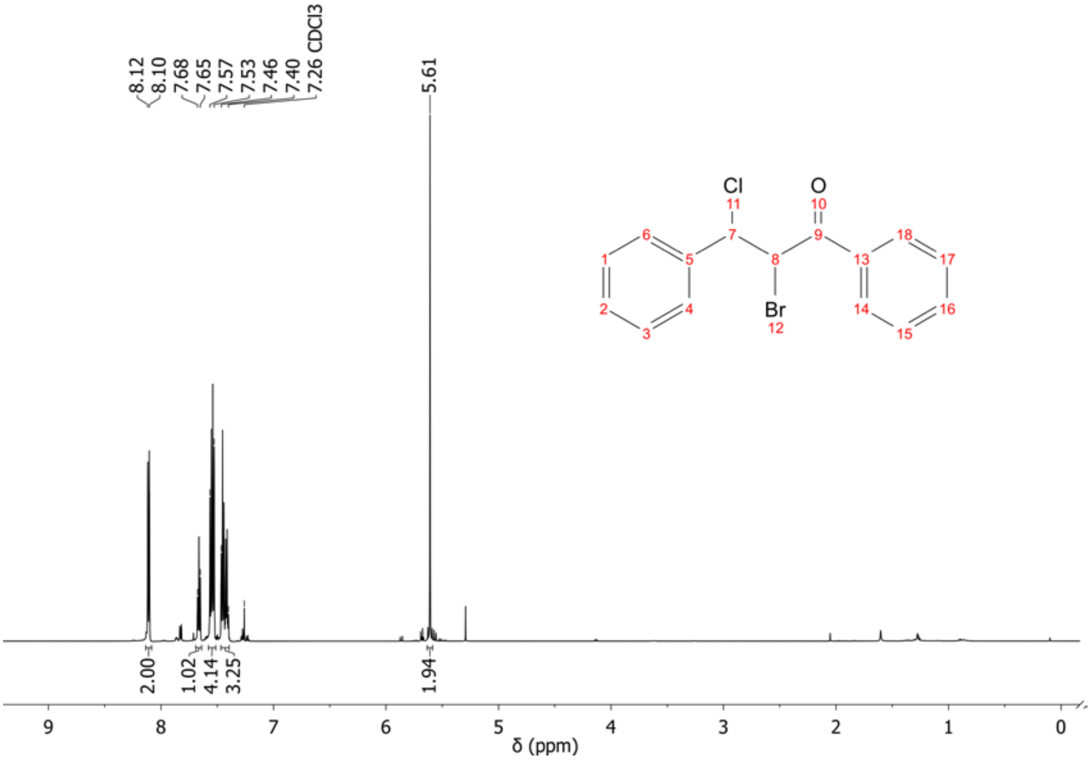

Figure S27. ^{1}H-NMR spectrum of the interhalogenation product **H**.

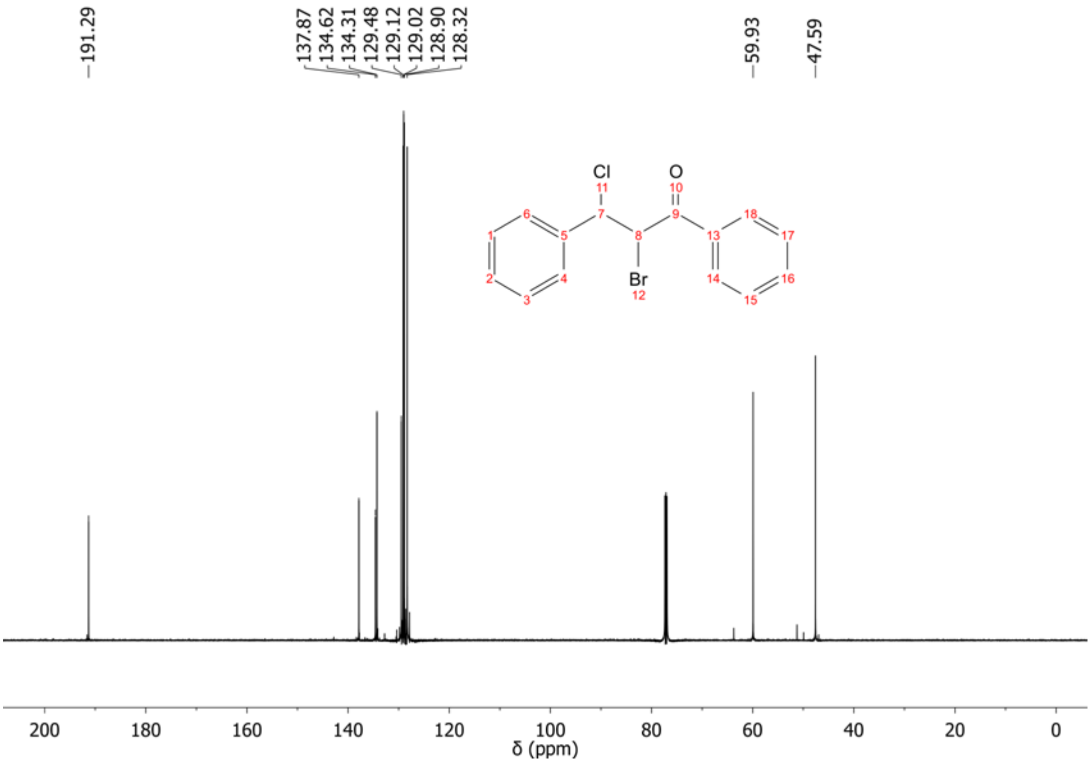

Figure S28. ^{13}C-NMR spectrum of the interhalogenation product **H**.

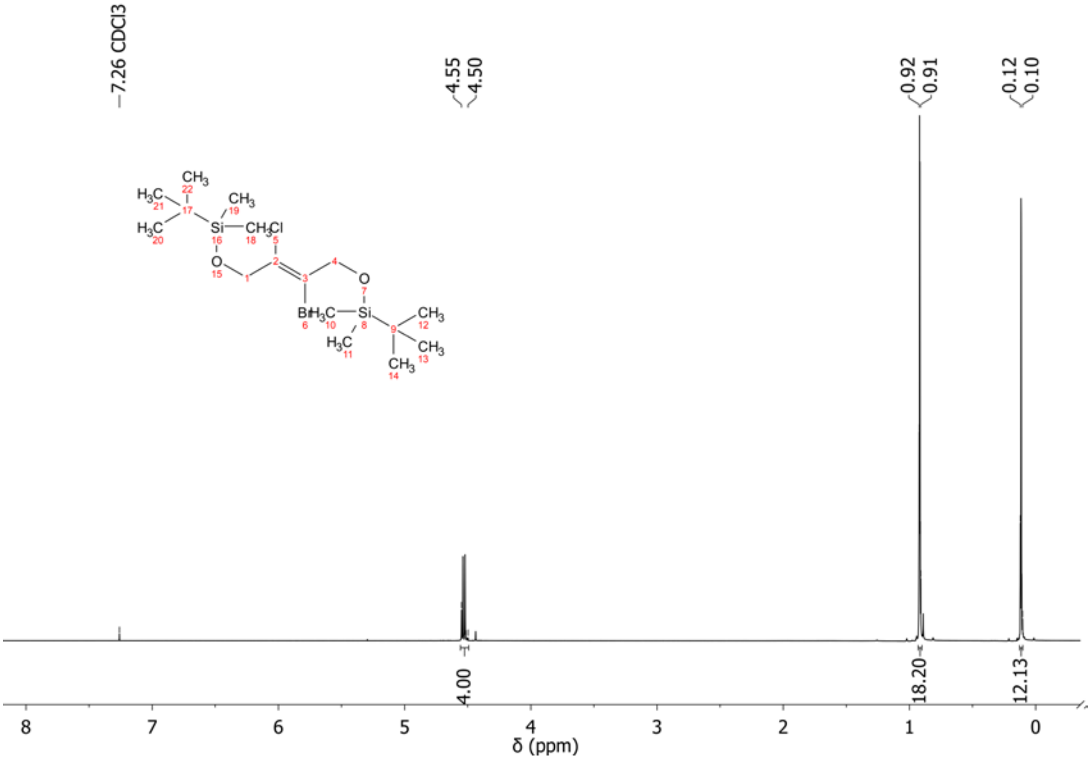

Figure S29. ^{1}H-NMR spectrum of the interhalogenation product **I**.

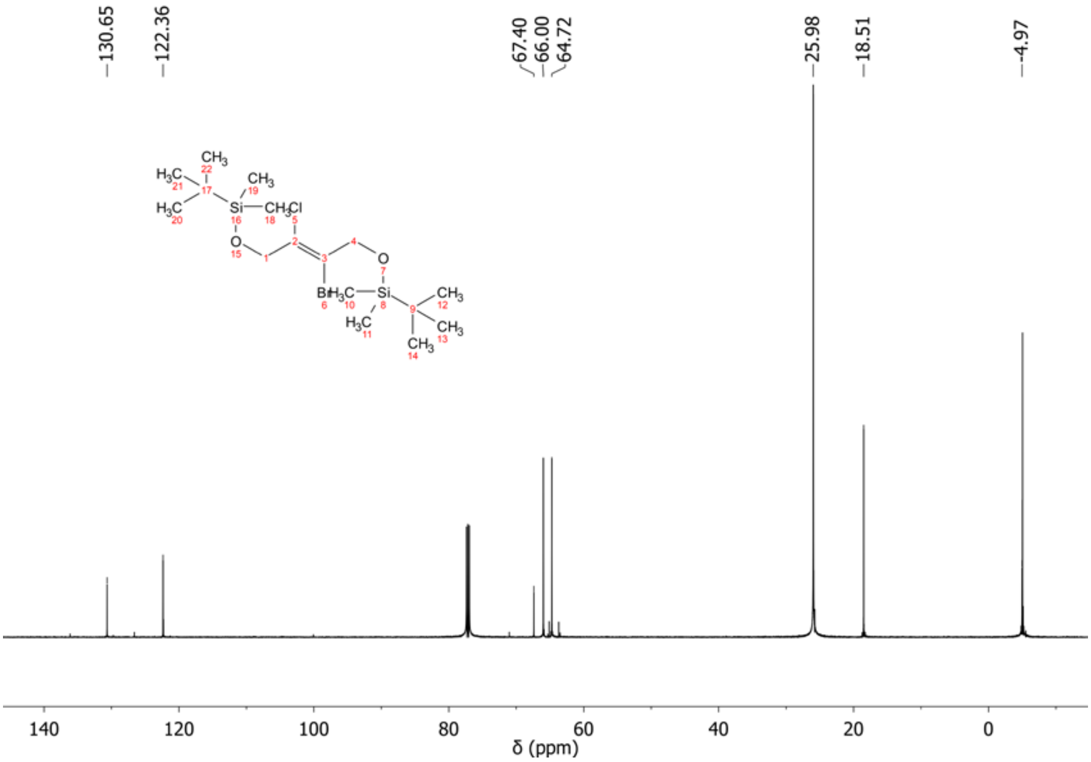

Figure S30. ^{13}C-NMR spectrum of the interhalogenation product **I**.

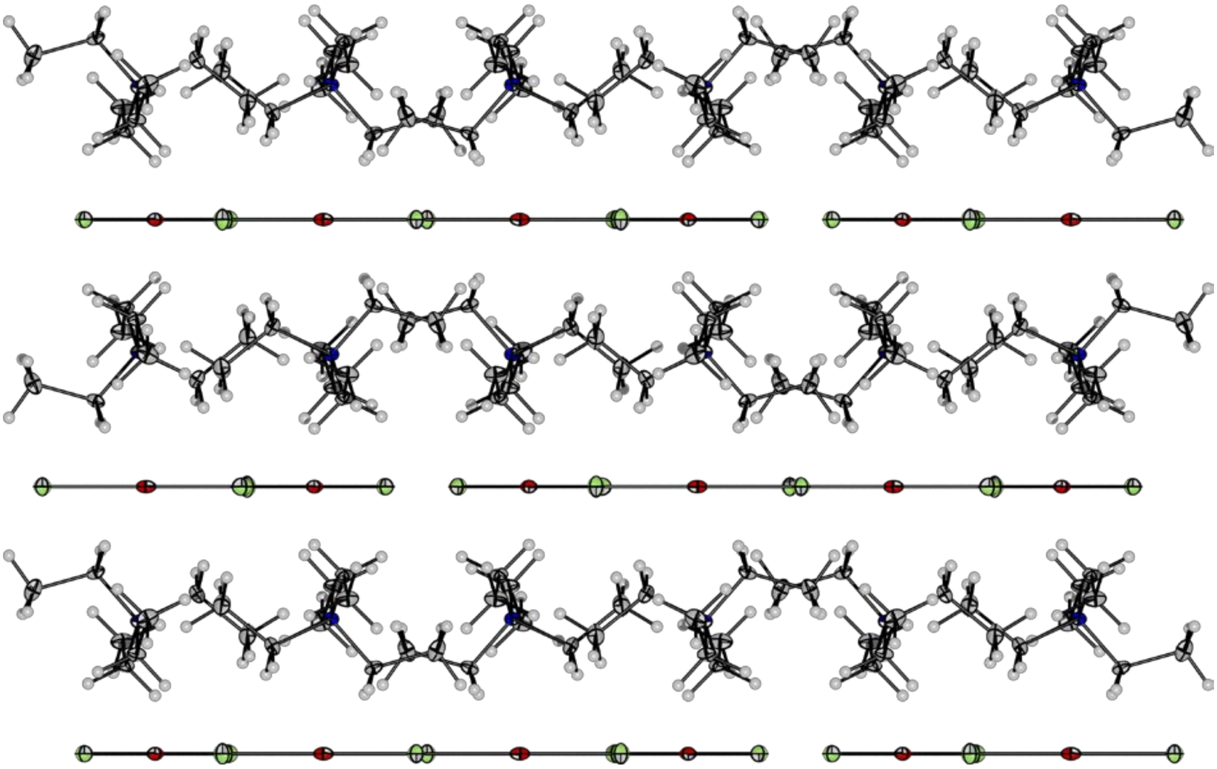

Figure S31. Crystal packing of [NEt_4][Cl(BrCl)] viewing along the crystallographic *x*-axis; thermal ellipsoids are shown with 50 % probability. The anions and cations are arranged in layers. There are no significant interactions (distance shorter than the sum of the van der Waals radii) between the anions or anion and hydrogen atoms.

h. Crystal Packing / Intermolecular interactions

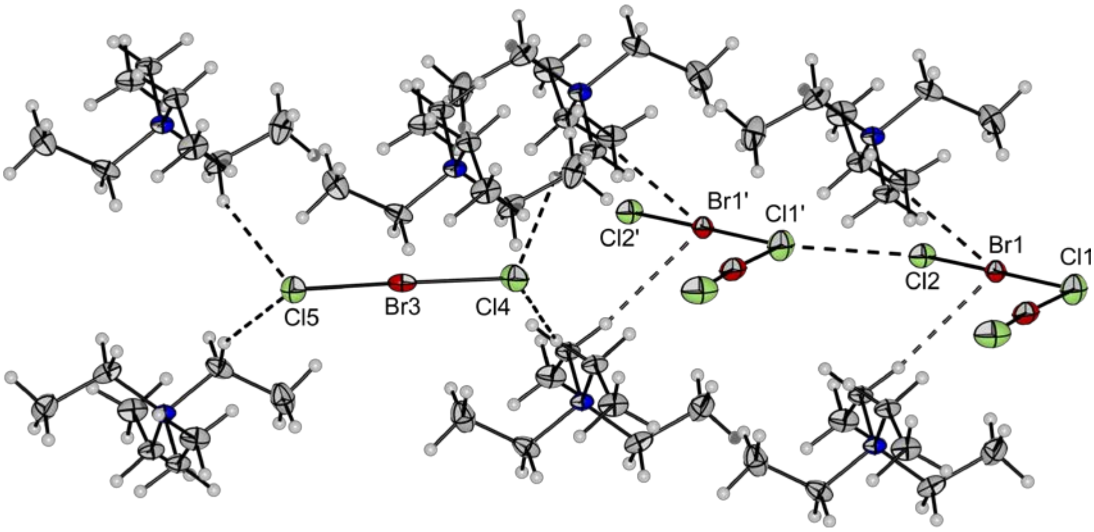

Figure S32. Crystal packing of [NEt_4]$_2$[Cl(BrCl)$_2$][ClBrCl]; thermal ellipsoids are shown with 50 % probability. The anions and cations are arranged in layers. There are weak hydrogen bond interactions to the anions (*d* H-Cl: 275 – 283 pm, *d* H-Br: 288 pm; depicted as dashed bonds) The [Cl(BrCl)$_2$]$^-$ units interact with each other, forming a chain (*d*. Cl2-Cl1' 318.1(1)).

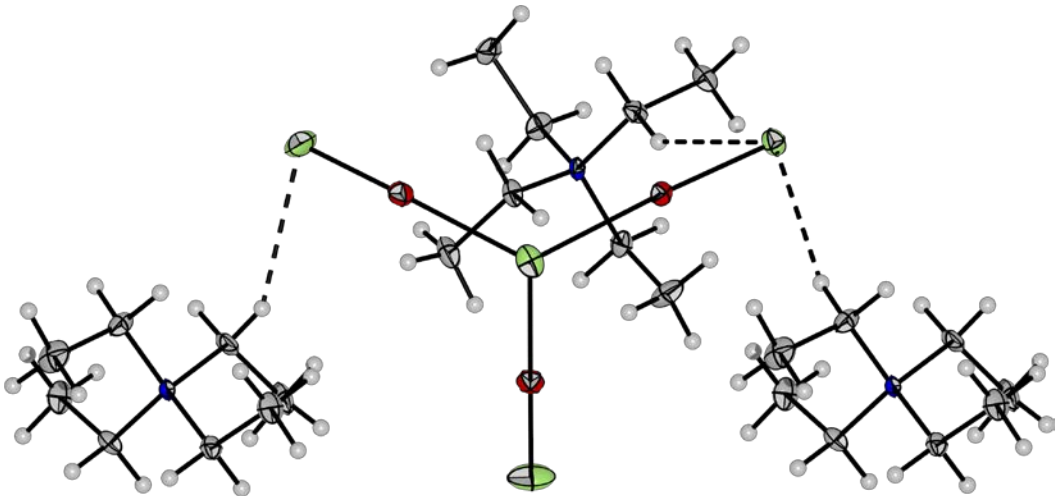

Figure S33. Hydrogen bonding between the $[Cl(BrCl)_3]^-$ anion and the surrounding $[NEt_4]^+$ cations; thermal ellipsoids are shown with 50 % probability. Three weak hydrogen bonds to the terminal chlorine atoms are observed (*d* H-Cl: 270 – 284 pm; depicted as dashed bonds). There are no interactions between the anions.

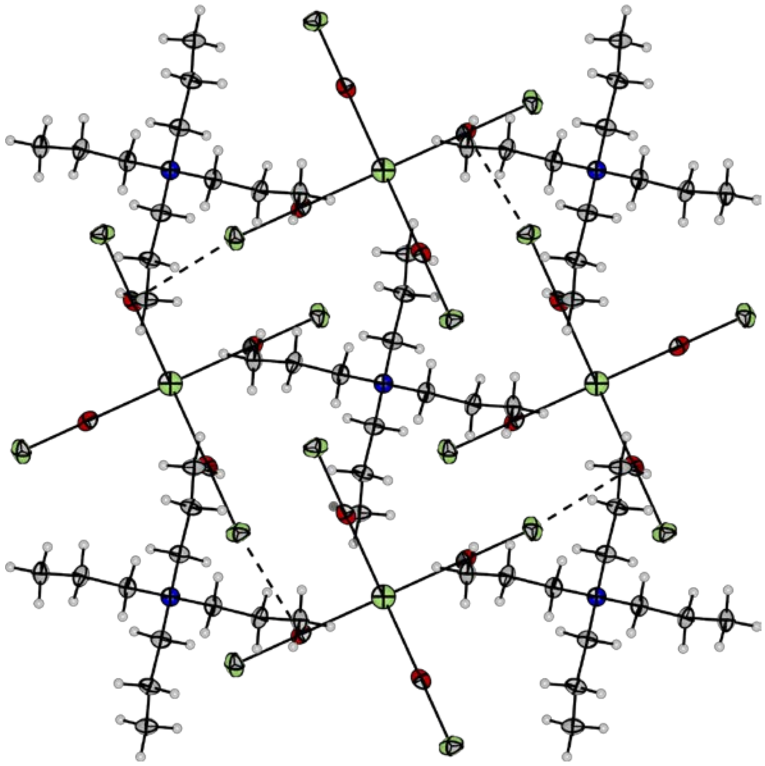

Figure S34. Crystal packing of $[NPr_4][Cl(BrCl)_4]$ viewing along the crystallographic *z*-axis; thermal ellipsoids are shown with 50 % probability. The anions and cations are arranged in columns. The intermolecular distances are depicted as dashed bonds (*d* Br-Cl: 350.8(2) pm). There are no interactions with the cations.

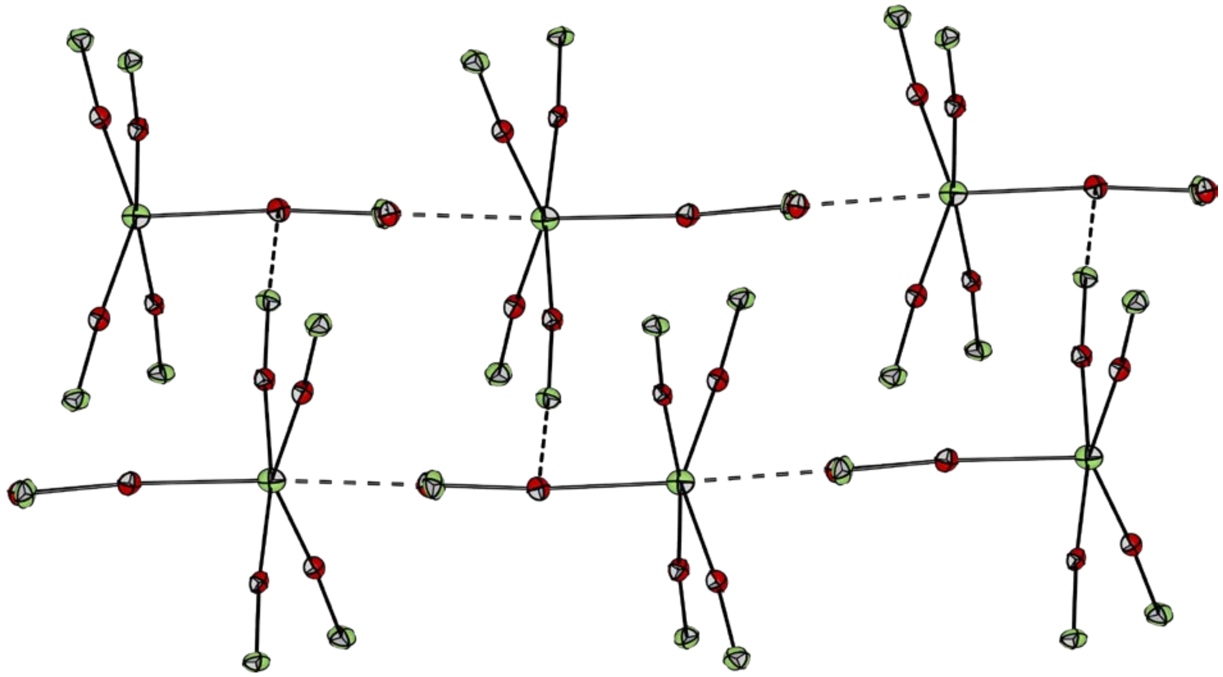

Figure S35. Anion-anion interactions of the $[Cl(BrCl)_5]^-$ units; thermal ellipsoids are shown with 50 % probability. A Br_2 molecule interacts with two central chlorides (*d* Br-Cl: 326.7(5), 298.6(2) pm) to form an infinite chain. The chains are very weakly interconnected to form an extended network (*d* Br-Cl: 336.9(1) pm). There are no interactions with the cations.

i. Long Term Stability Studies

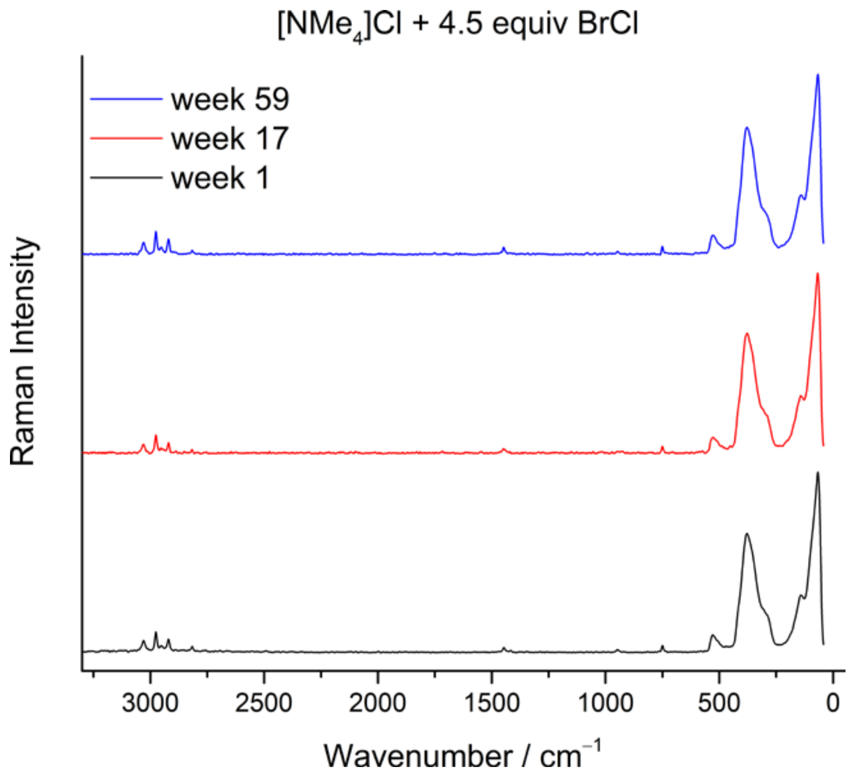

Figure S36. Long term stability studies of $[NMe_4]Cl$ and 4.5 equivalents of BrCl. After 59 weeks no new bands can be observed, which can be assigned to C-Br or C-Cl stretches. This indicates that the $[NMe_4]^+$ cation is stable against interhalogenation.

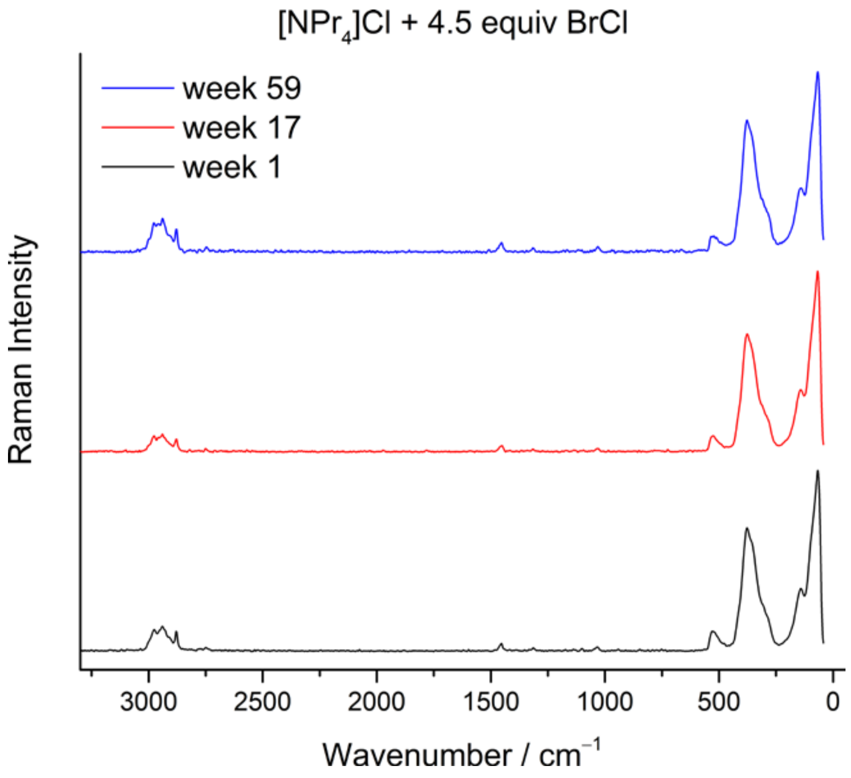

Figure S37. Long term stability studies of $[NPr_4]Cl$ and 4.5 equivalents of BrCl. After 59 weeks no new bands can be observed, which can be assigned to C-Br or C-Cl stretches. This indicates that the $[NPr_4]^+$ cation is stable against interhalogenation.

j. Computed Vibrational Frequencies

Table S5. Computed vibrational frequencies of $[Cl(BrCl)]^{-}$ in $D_{\infty h}$ symmetry.

B3LYP-D3(BJ)/def2-TZVPP					SCS-MP2/def2-TZVPP		
Nr.	Symmetry	Wavenumber [cm^{-1}]	IR Intensity [km mol^{-1}]	Nr.	Symmetry	Wavenumber [cm^{-1}]	IR Intensity [km mol^{-1}]
1	Π_u	117.0	3	1	Π_u	129.5	3
2	Π_u	117.0	3	2	Π_u	129.5	3
3	Σ_u^-	232.4	268	3	Σ_u^-	246.1	376
4	Σ_g^+	244.2	0	4	Σ_g^+	269.7	30

Table S6. Computed vibrational frequencies of $[Cl(BrCl)_2]^{-}$ in C_{2v} symmetry.

B3LYP-D3(BJ)/def2-TZVPP					SCS-MP2/def2-TZVPP		
Nr.	Symmetry	Wavenumber [cm^{-1}]	IR Intensity [km mol^{-1}]	Nr.	Symmetry	Wavenumber [cm^{-1}]	IR Intensity [km mol^{-1}]
1	A_1	20.3	0	1	A_1	18.7	0
2	B_1	95.5	4	2	B_1	99.7	24
3	A_2	98.5	0	3	A_2	107.8	0
4	A_1	105.2	1	4	A_1	109.3	2
5	B_2	120.2	2	5	B_2	131.7	3
6	A_1	187.6	25	6	B_1	167.0	502
7	B_1	190.6	346	7	A_1	193.7	48
8	B_1	283.7	165	8	B_1	313.9	142
9	A_1	305.7	50	9	A_1	335.0	63

Table S7. Computed vibrational frequencies of $[Cl(BrCl)_3]^{-}$ in C_{3v} symmetry.

B3LYP-D3(BJ)/def2-TZVPP					SCS-MP2/def2-TZVPP		
Nr.	Symmetry	Wavenumber [cm^{-1}]	IR Intensity [km mol^{-1}]	Nr.	Symmetry	Wavenumber [cm^{-1}]	IR Intensity [km mol^{-1}]
1	E	13.5	0	1	A_1	16.2	0
2	E	13.5	0	2	E	16.5	0
3	A_1	17.0	0	3	E	16.5	0
4	E	86.6	6	4	E	86.9	25
5	E	86.6	6	5	E	86.9	25
6	A_2	87.5	0	6	A_1	93.3	1
7	A_1	90.5	0	7	A_2	93.8	0
8	E	95.8	5	8	E	98.6	4
9	E	95.8	5	9	E	98.6	4
10	A_1	157.6	7	10	E	157.5	196
11	E	173.0	166	11	E	157.5	196
12	E	173.0	166	12	A_1	164.8	18
13	E	316.0	173	13	E	350.3	143
14	E	316.0	173	14	E	350.3	143
15	A_1	342.3	20	15	A_1	373.3	28

Table S8. Computed vibrational frequencies of $[Cl(BrCl)_4]^-$ in T_d symmetry.

B3LYP-D3(BJ)/def2-TZVPP				SCS-MP2/def2-TZVPP			
Nr.	Symmetry	Wavenumber [cm^{-1}]	IR Intensity [km mol^{-1}]	Nr.	Symmetry	Wavenumber [cm^{-1}]	IR Intensity [km mol^{-1}]
1	E	7.9	0	1	E	6.9	0
2	E	7.9	0	2	E	6.9	0
3	T_2	9.1	0	3	T_2	9.1	0
4	T_2	9.1	0	4	T_2	9.1	0
5	T_2	9.1	0	5	T_2	9.1	0
6	A_1	75.6	0	6	A_1	73.0	0
7	T_2	78.7	10	7	T_2	78.5	16
8	T_2	78.7	10	8	T_2	78.5	16
9	T_2	78.7	10	9	T_2	78.5	16
10	T_1	80.2	0	10	T_1	85.9	0
11	T_1	80.2	0	11	T_1	85.9	0
12	T_1	80.2	0	12	T_1	85.9	0
13	E	90.5	0	13	E	93.0	0
14	E	90.5	0	14	E	93.0	0
15	T_2	170.8	104	15	T_2	163.9	122
16	T_2	170.8	104	16	T_2	163.9	122
17	T_2	170.8	104	17	T_2	163.9	122
18	T_2	338.0	155	18	T_2	374.1	124
19	T_2	338.0	155	19	T_2	374.1	124
20	T_2	338.0	155	20	T_2	374.1	124
21	A_1	363.1	0	21	A_1	393.8	0

Table S9. Computed vibrational frequencies of $[Cl(BrCl)_5]^-$ in D_{3h} symmetry.

B3LYP-D3(BJ)/def2-TZVPP				SCS-MP2/def2-TZVPP			
Nr.	Symmetry	Wavenumber [cm^{-1}]	IR Intensity [km mol^{-1}]	Nr.	Symmetry	Wavenumber [cm^{-1}]	IR Intensity [km mol^{-1}]
1	E'	6.2	0	1	E'	14.6	0
2	E'	6.2	0	2	E'	14.6	0
3	E'	16.1	0	3	E''	15.5	0
4	E'	16.1	0	4	E''	15.5	0
5	E''	18.4	0	5	A_2''	22.5	0
6	E''	18.4	0	6	E'	25.5	0
7	A_2''	19.1	0	7	E'	25.5	0
8	A_1'	52.0	0	8	A_1'	51.0	0
9	E'	67.0	1	9	A_1'	68.9	0
10	E'	67.0	1	10	E'	76.1	9
11	E''	67.5	0	11	E'	76.1	9
12	E''	67.5	0	12	E''	81.7	0
13	A_1'	70.6	0	13	E''	81.7	0

14	A_2''	75.1	7	14	E'	82.5	0
15	E'	75.7	9	15	E'	82.5	2
16	E'	75.7	9	16	E'	82.5	2
17	A_2'	75.9	0	17	A_2'	83.3	10
18	E''	81.9	0	18	E''	92.1	0
19	E''	81.9	0	19	E''	92.1	0
20	A_2''	139.0	153	20	A_2''	150.5	153
21	E'	157.4	100	21	E'	155.6	105
22	E'	157.4	100	22	E'	155.6	105
23	E'	353.7	142	23	E'	390.3	104
24	E'	353.7	142	24	E'	390.3	104
25	A_1'	359.7	0	25	A_1'	394.0	0
26	A_2''	368.6	152	26	A_2''	399.4	115
27	A_1'	383.9	0	27	A_1'	411.2	0

Table S10. Computed vibrational frequencies of $[Cl(BrCl)_6]^-$ in O_h symmetry.

B3LYP-D3(BJ)/def2-TZVPP					SCS-MP2/def2-TZVPP		
Nr.	Symmetry	Wavenumber [cm^{-1}]	IR Intensity [km mol^{-1}]	Nr.	Symmetry	Wavenumber [cm^{-1}]	IR Intensity [km mol^{-1}]
1	T_{2u}	11.1	0	1	T_{2u}	15.3	0
2	T_{2u}	11.1	0	2	T_{2u}	15.3	0
3	T_{2u}	11.1	0	3	T_{2u}	15.3	0
4	T_{2g}	17.0	0	4	T_{2g}	20.6	0
5	T_{2g}	17.0	0	5	T_{2g}	20.6	0
6	T_{2g}	17.0	0	6	T_{2g}	20.6	0
7	T_{1u}	17.1	0	7	T_{1u}	27.4	0
8	T_{1u}	17.1	0	8	T_{1u}	27.4	0
9	T_{1u}	17.1	0	9	T_{1u}	27.4	0
10	E_g	51.8	0	10	E_g	53.3	0
11	E_g	51.8	0	11	E_g	53.3	0
12	T_{1g}	64.0	0	12	A_{1g}	67.5	0
13	T_{1g}	64.0	0	13	T_{1u}	77.3	5
14	T_{1g}	64.0	0	14	T_{1u}	77.3	5
15	T_{2u}	64.7	0	15	T_{1u}	77.3	5
16	T_{2u}	64.7	0	16	T_{1g}	79.2	0
17	T_{2u}	64.7	0	17	T_{1g}	79.2	0
18	T_{1u}	66.6	3	18	T_{1g}	79.2	0
19	T_{1u}	66.6	3	19	T_{2u}	80.2	0
20	T_{1u}	66.6	3	20	T_{2u}	80.2	0
21	A_{1g}	67.4	0	21	T_{2u}	80.2	0
22	T_{2g}	73.7	0	22	T_{2g}	86.3	0
23	T_{2g}	73.7	0	23	T_{2g}	86.3	0
24	T_{2g}	73.7	0	24	T_{2g}	86.3	0

25	T_{1u}	137.6	136	25	T_{1u}	151.6	125
26	T_{1u}	137.6	136	26	T_{1u}	151.6	125
27	T_{1u}	137.6	136	27	T_{1u}	151.6	125
28	T_{1u}	373.0	135	28	T_{1u}	405.6	95
29	T_{1u}	373.0	135	29	T_{1u}	405.6	95
30	T_{1u}	373.0	135	30	T_{1u}	405.6	95
31	E_g	373.3	0	31	E_g	405.8	0
32	E_g	373.3	0	32	E_g	405.8	0
33	A_{1g}	393.1	0	33	A_{1g}	419.7	0

k. Additional Information

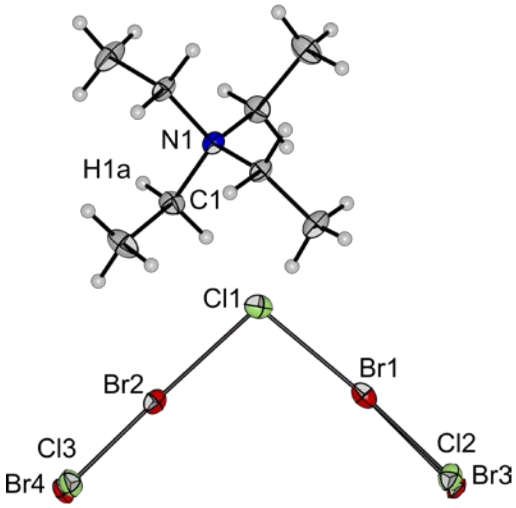

Figure S38. Molecular structure of $[NEt_4][Cl(BrCl)_2]$ in the solid state with thermal ellipsoids set at 50 % probability. Selected bond lengths [pm] and angles [°]: Cl1-Br1 259.6(1), Cl1-Br2 265.5(1), Br1-Cl2 224.4(3), Br2-Cl3 220.7(4), Br1-Br3 247.0(9), Br2-Br4 241.1(7); Cl1-Br1-Cl2 175.0(2), Cl1-Br2-Cl3 176.1(2), Br2-Cl1-Br1 97.1(1); population of the disorders: Cl2: 88%, Br3: 12 %, Cl3: 81 %, Br4: 19 %.

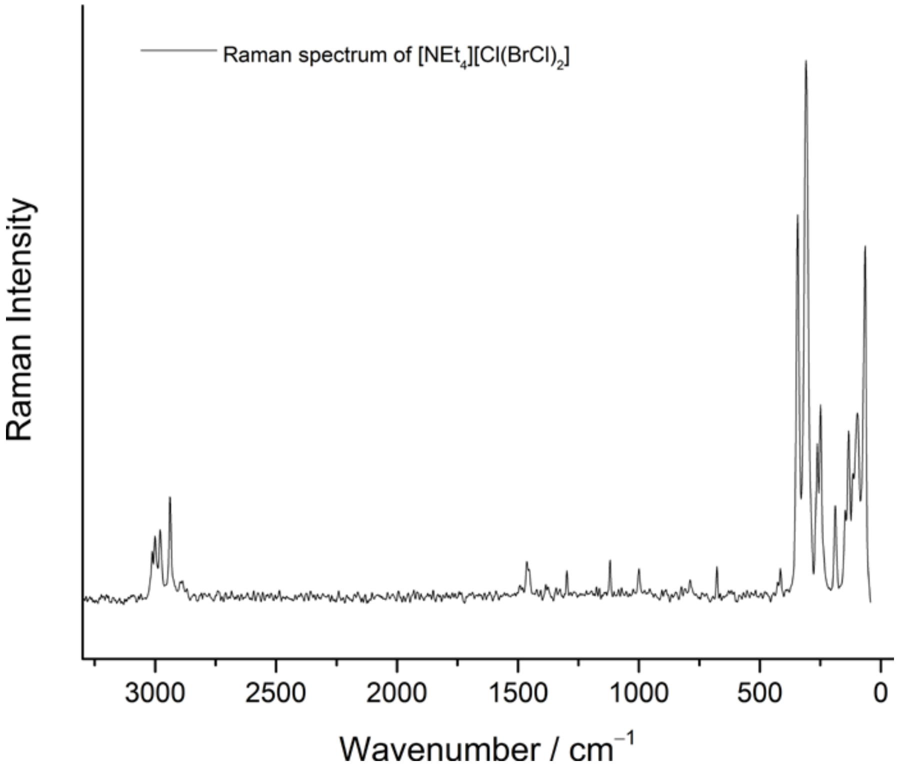

Figure S39. Raman spectrum of a single crystal of $[NEt_4][Cl(BrCl)_2]$, taken at low temperature (–196 °C).

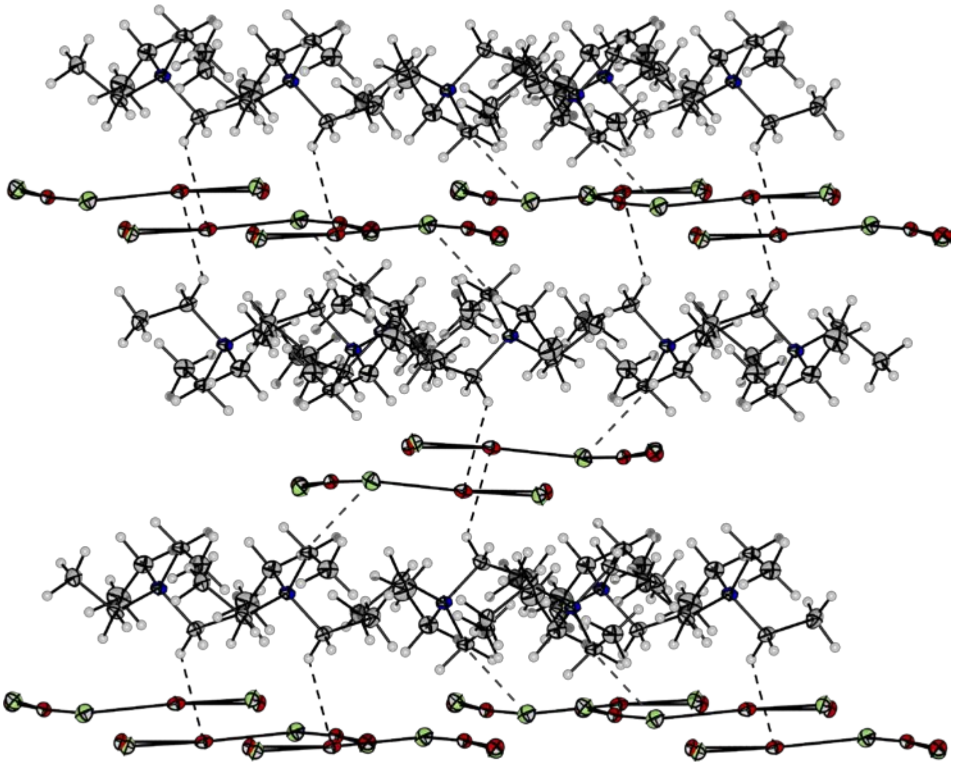

Figure S40. Crystal packing of $[NEt_4][Cl(BrCl)_2]$; thermal ellipsoids are shown with 50 % probability. The anions and cations are arranged in layers. There are very weak hydrogen bond interactions to the anions (*d* H-Cl: 279 pm, *d* H-Br: 286 pm; depicted as dashed bonds) Anion-anion interactions are depicted in Figure S41.

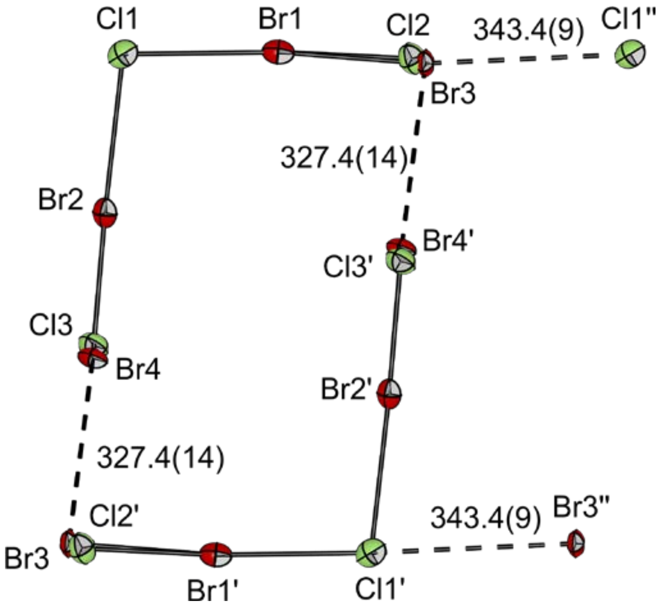

Figure S41. Anion-anion interactions of the $[Cl(BrCl)_2]^-$ units viewing along the crystallographic *y*-axis; thermal ellipsoids are shown with 50 % probability. Two pentahalide anions form a rectangle and are connected via the bromine atoms (*d* Br3-Br4: 327.4(14) pm). The connections between Br and Cl are less pronounced (*d* Cl2-Br4': 334.0(8) pm, *d* Cl3-Br3' 350.3(12)). The rectangles are interconnected via the Cl1'-Br3''' interaction (343.4(9) pm).

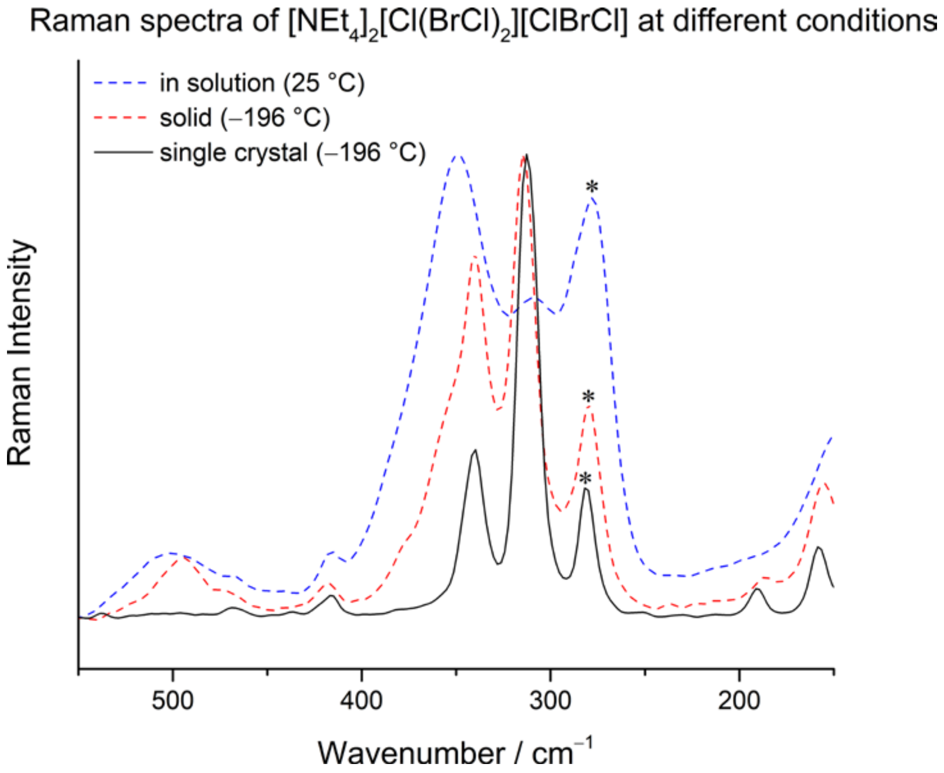

Figure S42. Raman spectra of $[NEt_4]_2[Cl(BrCl)_2][ClBrCl]$ at different conditions. The spectra were taken in a DCM solution at 25 °C, as solid (–196 °C) and as single crystal Raman scope spectrum (–196 °C). Gas phase calculations show that the symmetric stretching mode (A_1) of the coordinating BrCl molecules has a higher Raman intensity (see Figure 6) than the asymmetric stretch (B_1). In solution, these intensities are in agreement with the calculations. However, spectra of the solid state show, that the intensities change, resulting in a higher intensity for the asymmetric stretching mode. This can be explained by interactions in the solid state. The band at 281 cm^{-1} associated with $[ClBrCl]^-$ is indicated by asterisks.

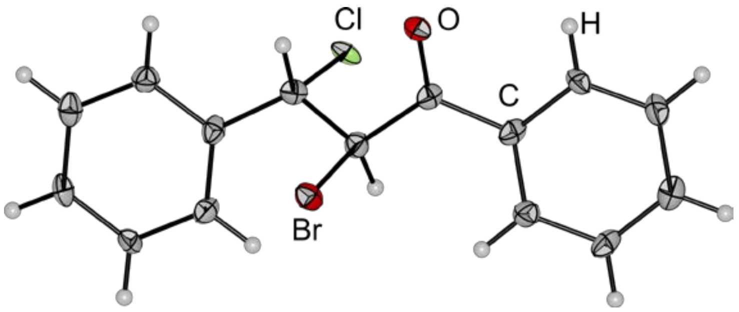

Figure S43. Solid state structure of the product **H** after interhalogenation with the BrCl based reactive ionic liquid; thermal ellipsoids are shown with 50 % probability. The halogens are arranged trans to each other.

I. xyz-Files of the Calculated Molecules

$[Cl(BrCl)]^{–}$

B3LYP-D3(BJ)/def2-TZVPP:

0.00000000000000	0.00000000000000	-4.65478608806984	cl
0.00000000000000	0.00000000000000	-0.00000000000000	br
0.00000000000000	0.00000000000000	4.65478608806984	cl

E_{tot} = -3494.476772972 H, *ZPE* = 4.214 kJ/mol, *H* = 19.25 kJ/mol, μ = -66.39 kJ/mol

SCS-MP2/def2-TZVPP:

0.00000000000000	0.00000000000000	-4.56717617284753	cl
0.00000000000000	0.00000000000000	0.00000000000000	br
0.00000000000000	0.00000000000000	4.56717617284753	cl

$E_{(SCF)}$ = -3491.495333171 H, $E_{(MP2)}$ = -1.026591420411 H, *ZPE* = 4.634 kJ/mol

H = 19.38 kJ/mol, μ = -65.25 kJ/mol

$[Cl(BrCl)_2]^{–}$

B3LYP-D3(BJ)/def2-TZVPP:

0.00000000000000	0.00000000000000	-3.21138772166569	cl
-4.03201490368139	0.00000000000000	-0.40108236354168	br
4.03201490368139	0.00000000000000	-0.40108236354168	br
7.71939332291606	0.00000000000000	2.00671238810105	cl
-7.71939332291606	0.00000000000000	2.00671238810105	cl

E_{tot} = -6528.696640102 H, *ZPE* = 8.345 kJ/mol, *H* = 33.57 kJ/mol, μ = -92.22 kJ/mol

SCS-MP2/def2-TZVPP:

0.00000000000000	0.00000000000000	-3.34336507817772	cl
-3.91977218528769	0.00000000000000	-0.41982630251125	br
3.91977218528769	0.00000000000000	-0.41982630251125	br
7.41698127141964	0.00000000000000	2.09144500532661	cl
-7.41698127141964	0.00000000000000	2.09144500532661	cl

$E_{(SCF)}$ = -6523.408663161 H, $E_{(MP2)}$ = -1.700585487184 H, *ZPE* = 8.851 kJ/mol

H = 33.76 kJ/mol, μ = -91.16 kJ/mol

$[Cl(BrCl)_3]^-$

B3LYP-D3(BJ)/def2-TZVPP:

-0.00000000000000	0.00000000000000	-2.38862142703802	cl
-2.34937161747260	4.06923100732284	-0.39846634525948	br
-2.34937161747260	-4.06923100732284	-0.39846634525948	br
4.69874323494525	0.00000000000000	-0.39846634525948	br
8.68845360089481	0.00000000000000	1.20131512417996	cl
-4.34422680044738	7.52442153797727	1.20131512417996	cl
-4.34422680044738	-7.52442153797727	1.20131512417996	cl

E_{tot} = -9562.906293914 H, *ZPE* = 12.24 kJ/mol, *H* = 49.17 kJ/mol, μ = -115.05 kJ/mol

SCS-MP2/def2-TZVPP:

-0.00000000000000	0.00000000000000	-2.71886913342799	cl
-2.29119276264963	3.96846227484326	-0.45817098245949	br
-2.29119276264963	-3.96846227484326	-0.45817098245949	br
4.58238552529929	0.00000000000000	-0.45817098245949	br
8.37584103748935	0.00000000000000	1.37110233017664	cl
-4.18792051874466	7.25369111652598	1.37110233017664	cl
-4.18792051874466	-7.25369111652598	1.37110233017664	cl

$E_{(SCF)}$ = -9555.321018629 H, $E_{(MP2)}$ = -2.369837219305 H, *ZPE* = 12.93 kJ/mol

H = 49.48 kJ/mol, μ = -112.71 kJ/mol

$[Cl(BrCl)_4]^-$

B3LYP-D3(BJ)/def2-TZVPP:

-0.00000000000000	0.00000000000000	-0.00000000000000	cl
-3.01884831384178	-3.01884831384178	3.01884831384178	br
3.01884831384178	-3.01884831384178	-3.01884831384178	br
-3.01884831384178	3.01884831384178	-3.01884831384178	br
3.01884831384178	3.01884831384178	3.01884831384178	br
-5.46944696218501	-5.46944696218501	5.46944696218501	cl
5.46944696218501	5.46944696218501	5.46944696218501	cl
-5.46944696218501	5.46944696218501	-5.46944696218501	cl
5.46944696218501	-5.46944696218501	-5.46944696218501	cl

E_{tot} = -12597.11120670 H, *ZPE* = 15.81 kJ/mol, *H* = 64.82 kJ/mol, μ = -140.55 kJ/mol

SCS-MP2/def2-TZVPP:

-0.00000000000000	0.00000000000000	0.00000000000000	cl
-3.03262051540363	-3.03262051540363	3.03262051540363	br
3.03262051540363	-3.03262051540363	-3.03262051540363	br
-3.03262051540363	3.03262051540363	-3.03262051540363	br
3.03262051540363	3.03262051540363	3.03262051540363	br
-5.43875660644968	-5.43875660644968	5.43875660644968	cl
5.43875660644968	5.43875660644968	5.43875660644968	cl
-5.43875660644968	5.43875660644968	-5.43875660644968	cl
5.43875660644968	-5.43875660644968	-5.43875660644968	cl

$E_{(SCF)}$ = -12587.23284961 H, $E_{(MP2)}$ = -3.036569955557 H, *ZPE* = 16.76 kJ/mol

H = 65.28 kJ/mol, μ = -139.27 kJ/mol

$[Cl(BrCl)_5]^-$

B3LYP-D3(BJ)/def2-TZVPP:

-0.00000000000000	0.00000000000000	-0.00000000000000	cl
-2.67580366236152	-4.63462789428903	0.00000000000000	br
-2.67580366236152	4.63462789428903	0.00000000000000	br
5.35160732472303	0.00000000000000	0.00000000000000	br
0.00000000000000	0.00000000000000	5.48805052183008	br
0.00000000000000	0.00000000000000	-5.48805052183008	br
9.56402041542057	0.00000000000000	0.00000000000000	cl
-4.78201020771027	-8.28268464206720	0.00000000000000	cl
-4.78201020771027	8.28268464206720	0.00000000000000	cl
0.00000000000000	0.00000000000000	9.67207751604432	cl
0.00000000000000	0.00000000000000	-9.67207751604432	cl

E_{tot} = -15631.31125760 H, *ZPE* = 19.16 kJ/mol, *H* = 80.46 kJ/mol, μ = -162.28 kJ/mol

SCS-MP2/def2-TZVPP:

0.00000000000000	0.00000000000000	0.00000000000000	cl
-2.68812454489377	-4.65596828882895	0.00000000000000	br
-2.68812454489377	4.65596828882895	0.00000000000000	br
5.37624908978751	0.00000000000000	0.00000000000000	br
0.00000000000000	0.00000000000000	5.45552697524794	br
0.00000000000000	0.00000000000000	-5.45552697524794	br
9.51781151670752	0.00000000000000	0.00000000000000	cl
-4.75890575835375	-8.24266656190080	0.00000000000000	cl
-4.75890575835375	8.24266656190080	0.00000000000000	cl
0.00000000000000	0.00000000000000	9.58271041987689	cl
0.00000000000000	0.00000000000000	-9.58271041987689	cl

$E_{(SCF)}$ = -15619.13933117 H, $E_{(MP2)}$ = -3.706321438418 H, *ZPE* = 21.12 kJ/mol

H = 81.14 kJ/mol, μ = -151.05 kJ/mol

$[Cl(BrCl)_6]^-$

B3LYP-D3(BJ)/def2-TZVPP:

-0.00000000000000	-0.00000000000000	0.00000000000000	cl
5.51151017729707	0.00000000000000	-0.00000000000000	br
-0.00000000000000	-0.00000000000000	-5.51151017729707	br
0.00000000000000	-5.51151017729707	0.00000000000000	br
0.00000000000000	0.00000000000000	5.51151017729707	br
-5.51151017729707	-0.00000000000000	0.00000000000000	br
-0.00000000000000	5.51151017729707	0.00000000000000	br
-0.00000000000000	9.68747412917864	0.00000000000000	cl
9.68747412917864	0.00000000000000	-0.00000000000000	cl
0.00000000000000	-0.00000000000000	-9.68747412917864	cl
-0.00000000000000	0.00000000000000	9.68747412917864	cl
-9.68747412917864	-0.00000000000000	0.00000000000000	cl
0.00000000000000	-9.68747412917864	-0.00000000000000	cl

E_{tot} = -18665.51215904 H, *ZPE* = 22.44 kJ/mol, *H* = 96.12 kJ/mol, μ = -181.05 kJ/mol

SCS-MP2/def2-TZVPP:

-0.00000000000000	-0.00000000000000	-0.00000000000000	cl
5.49514970038369	0.00000000000000	0.00000000000000	br
0.00000000000000	-0.00000000000000	-5.49514970038369	br
0.00000000000000	-5.49514970038369	-0.00000000000000	br
-0.00000000000000	0.00000000000000	5.49514970038369	br
-5.49514970038369	-0.00000000000000	-0.00000000000000	br
-0.00000000000000	5.49514970038369	0.00000000000000	br
-0.00000000000000	9.61346003715582	0.00000000000000	cl
9.61346003715582	0.00000000000000	-0.00000000000000	cl
0.00000000000000	-0.00000000000000	-9.61346003715582	cl
-0.00000000000000	0.00000000000000	9.61346003715582	cl
-9.61346003715582	-0.00000000000000	0.00000000000000	cl
0.00000000000000	-9.61346003715582	0.00000000000000	cl

$E_{(SCF)}$ = -18651.04434211 H, $E_{(MP2)}$ = -4.378111120132 H, *ZPE* = 25.33 kJ/mol

H = 97.01 kJ/mol, μ = -165.00 kJ/mol
